# Recent Advances in Biomedical Photonic Sensors: A Focus on Optical-Fibre-Based Sensing

**DOI:** 10.3390/s21196469

**Published:** 2021-09-28

**Authors:** Mario Ochoa, José Francisco Algorri, Pablo Roldán-Varona, Luis Rodríguez-Cobo, José Miguel López-Higuera

**Affiliations:** 1 Photonics Engineering Group, University of Cantabria, 39005 Santander, Spain; algorrijf@unican.es (J.F.A.); pablo.roldan@unican.es (P.R.-V.); 2 Instituto de Investigación Sanitaria Valdecilla (IDIVAL), 39011 Santander, Spain; 3 CIBER-bbn, Institute of Health Carlos III, 28029 Madrid, Spain; luis.rodriguez@unican.es

**Keywords:** biomedical, photonic sensor, optical-fibre sensor, optical probes, catheter, needle, endoscope

## Abstract

In this invited review, we provide an overview of the recent advances in biomedical photonic sensors within the last five years. This review is focused on works using optical-fibre technology, employing diverse optical fibres, sensing techniques, and configurations applied in several medical fields. We identified technical innovations and advancements with increased implementations of optical-fibre sensors, multiparameter sensors, and control systems in real applications. Examples of outstanding optical-fibre sensor performances for physical and biochemical parameters are covered, including diverse sensing strategies and fibre-optical probes for integration into medical instruments such as catheters, needles, or endoscopes.

## 1. Introduction

The interaction between light and biological cells, tissues, organs, and body signals has enabled significant contributions of photonics to the medical field [1]. In particular, the possibility of sensing such interactions provides a way to enhance medical diagnosis, monitoring, treatments, and overall healthcare. In this context, biomedical photonic sensors play a crucial role.

Biomedical photonic sensors must be safe, highly stable, biocompatible, sterilization-compatible, free of recalibrations (especially in the short term), and meet other requirements when functioning in the clinical environment [1]. Furthermore, small size, robustness, and flexibility are highly desirable for the integration of devices into medical instruments (e.g., endoscopes, catheters). In particular, biomedical photonic sensors based on optical-fibre sensing have strong potential to meet such requirements. Optical fibres have additional key advantages for the medical field, such as miniaturization, immunity to electromagnetic radiation (e.g., for its use in magnetic resonance interventions), continuous monitoring, and, in some cases, the possibility of achieving higher sensitivities and accuracy than conventional sensing methods. Possibly, the most important differentiator of biomedical optical-fibre sensors is that they are flexible and small, thus suitable for integration into medical instruments such as catheters, needles, and endoscopes. There are many areas in which biomedical optical-fibre sensors have been investigated. For instance, physiological monitoring, minimally invasive surgery, biomechanics (or shape sensing), medical sensing related to cancers, infections, diseases, disorders, or physical and biochemical parameters determining health conditions [2,3,4,5,6].

In general, optical-fibre sensors are highly diverse. Since the optical properties of most materials are naturally responsive to their surroundings, there is a large number of sensing opportunities for optical-fibre sensors to measure several physical, chemical, and biological parameters [7]. Even though the modulation techniques of transducers are similar for most optical-fibre sensors (based on absorption, reflectance, fluorescence, and refractive index changes), the diversity of sensors keeps increasing. Physical alterations on the optical-fibre core, cladding, or end face, give rise to a large variety of sensing structures with different characteristics. Among the many that exist, it is worth highlighting optical structures based on gratings, either fibre Bragg gratings (and modified versions such as tilted or chirped) or long-period gratings; interferometry, standing out Mach–Zehnder (MZ), Michelson, Sagnac configurations, or Fabry–Pérot cavities, among others; or geometry-modified fibres, such as tapered and U-shaped fibres. Special coatings (or films) could also be applied to the fibres, for which metal coatings are the most used. These coatings provide, mainly, higher sensitivity to measurands by exploiting the surface plasmon resonance (SPR) effect. Recently, optical biosensors implementing biorecognition elements have enabled new functionalization and detection capabilities of the fibres. Furthermore, the potential of optical-fibre technology has been remarkably extended by combining optics and microfluidics (optofluidics [8,9]) using optical fibres, i.e., optical-fibre optofluidics [10,11]. Finally, another degree of flexibility (and diversity) on the design of optical-fibre sensors is imposed by the fibre type and its characteristics. For instance, single-mode, multimode, single-core, multicore, hollow-core, photonic-crystal fibres or polymer fibres. Overall, the potential of optical-fibre sensors is enormous, and a wide range of combinations may be employed.

**Figure 1:**
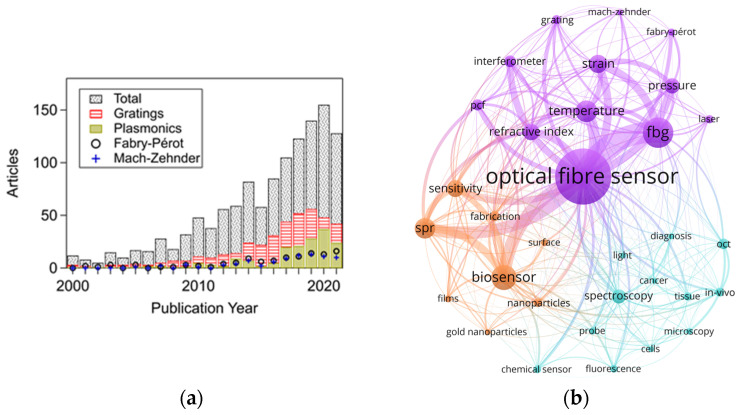
(**a**) Articles as a function of publication year retrieved by searching for optical-fibre sensors. Some representative types of optical-fibre sensors for biomedical applications are depicted. The data were taken from 1351 articles found in Web of Science Core Collection when searching for optical-fibre sensors (23,895 records) and restricting the results applicable to the biomedical field (accessed on 17 September 2021). (**b**) Map visualization of keywords links strength network using the same 1351 articles information. Only keywords exceeding twenty occurrences are depicted. The period selected is from 1965 to 2021. Abbreviations: magnetic resonance imaging: mri; surface plasmon resonance: spr; fibre Bragg grating: fbg; optical coherence tomography: oct; photonic crystal fibre: pcf. Graph created using VOSviewer software version 1.6.17 [13]. The evolution of published articles over the years and network maps of keywords related to optical-fibre sensors in general (not restricted to the biomedical field) can be found in the supporting information (Figures S1 and S2), including details on how these graphs were generated.

Presently, the application of optical-fibre sensors technology is increasing (Figure 1a) and progressively reaching maturity in specific biomedical applications [12]. In this regard, fibre Bragg grating or interferometric sensors (e.g., Fabry–Pérot cavities) are widely used to monitor physical parameters. In contrast, fluorescence-based, SPR-based (or exploiting evanescent field) sensors are primarily investigated to detect biological/chemical parameters. This is supported by Figure 1b, which shows a network map of keywords found in 1351 journal articles related to optical-fibre sensors for biomedical applications (for graphs regarding optical fibre sensors in general, see Figures S1 and S2 in the supporting information). In this map, the lines indicate a link between keywords. Thicker lines represent a stronger link between occurrences. Bigger circles correspond to a higher number of events for each keyword. In addition, three clusters are shown with different colours, for which each cluster groups a series of keywords sharing high linking strength between them. This map highlights the sensing techniques, parameters, etc., employed in the biomedical field over the years. Since a significantly higher number of publications correspond to the last decade (over 75% of the total), this decade dominates the keyword occurrences.

Although there has been an increasing interest in biomedical optical-fibre sensors, there is still a low level of implementation [6,14]. From the technical point of view, to widen the acceptance and implementation of the technology, optical-fibre sensors may require [15]: meeting a need other sensors cannot, showing outstanding performance at a similar cost of existing solutions, or significantly reducing their cost.

In this regard, optical-fibre sensors offer many possibilities to develop new medical devices to improve current methods. New technologies and the combination of two or more techniques may allow for enhanced capabilities and functionalities, for example, higher sensitivity, specificity to selected targets, and multiparameter sensing. On the other hand, general challenges still need to be considered: minimization of cross-sensitivities, increased resolution and dynamic range, enhanced stability and reliability, etc. [16]. Overall, technical innovations, remarkably superior performance, miniaturization, multiparameter sensing in a single fibre, and multiple sensors in a medical probe (including multiplexing), could show a route towards widespread implementation of biomedical optical-fibre sensors. The contribution presented here involves briefly many of these pathways.

This work reviews the latest advances in biomedical photonic sensors based on optical-fibre sensing. The examples described correspond to a selection of the most recent works (mainly within the last five years) intended to convey the advancement and novelty of biomedical optical-fibre sensors for measuring several biomedical parameters. Priority was given to publications dealing with real applications whereas most of the latest advances described are not covered in previous reviews.

This review is divided into three main sections. First, a conceptual section regarding biomedical photonic sensors is provided to establish the basic definition of what should be understood as a biomedical photonic sensor (Section 2). Then, Section 3 describes the most recent biomedical photonic sensors based on optical fibres and acting as stand-alone devices. This section describes the latest advancements in sensing physical parameters (vital signs and temperature), biochemical parameters (pH, oxygen, cancer-related and others), including a brief description of multiparameter sensors for lab-on-fibre implementation. Finally, Section 4 presents fibre-optical probes integrated into medical instruments such as endoscopes, catheters, and needles. It includes fibre-optical probes for spectroscopy and modified probes to sense shape and force.

For information about fundamentals, operation principles of the sensors, and previous works not covered in this review, the reader is referred to the literature regarding optical-fibre sensors applied in the biomedical field [6,12,17,18,19,20,21,22]. For an extended list of reviews of optical-fibre sensors focused on biomedical applications (or with biomedical interest), the reader is referred to Table S1 in the supporting information.

## 2. Biomedical Photonic Sensors

The detection, capture, measurement, supervision, and control of magnitudes of given objects are requirements of paramount importance in today’s times. Obtaining specific information about a given parameter or measurand (M_x_) in any state or domain on/in an object (O_y_) placed in a given environment is commonly recognized as a detection process. Sensors are the devices or systems developed to detect and capture physical, chemical, biological, biomedical measurands, etc., translating and reproducing them in the electrical domain to be useful in real applications in today’s world. Photonic Sensors (PS) are the set of devices/systems designed to carry out the faithful reproduction of the measurand in the electrical domain using photonic technologies in their key sensor parts [23].

In any photonic sensor, the light (L_x_) coming from the target/object (O_y_) includes the information concerning its specific measurand (M_x_), that, after being detected and processed, enables its faithful reproduction in the electric domain. The photons of the light, L_x_, from the O_y_ can be produced by the object itself or could be a consequence of their excitation with appropriate optical radiation or any other source of excitation energy. The light from the target includes information (modulated by the measurand or modulating signal) on several of its main characteristics such as amplitude, phase, frequency, polarization, or any other light characteristic [24,25].

**Figure 2:**
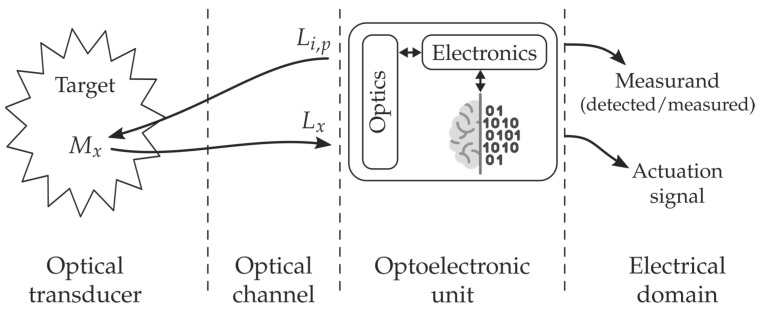
Illustration of the Biomedical Photonic Sensor concept and its conceptual block diagram. The photonic sensor provides representative and faithful electrical signals of physical, chemical, biological, or physiological measurands on/in a given object or target. When a BPS is equipped with some kind of intelligence and provides actuation signals, it is transformed into a Biomedical Smart Photonic Sensor (BSPS). It is integrated into three main parts: Optical Transducer; Optical Channel and Optoelectronic Unit [31,32,33]. Courtesy of the authors.

Three main parts or subsystems are identified by analysing the requirements to build a real PS: the transducer, the optical channel, and the optoelectronic unit, as depicted in Figure 2. The optical transducer is the part in which the measurand modulates the light; the optical channel is the part that optically connects the transducer with the optoelectronic unit, and the latter is the part in which the optical signal coming from the object (L_x_) is photodetected, amplified, demodulated, processed, and equalized, offering, as a result, an output electric signal (analogic or digital) which is a faithful reproduction of the measurand [24]. The optoelectronic unit also includes, if it is required, all technology concerning the optical source/s to interrogate or/and pump (L_i,p_) the object to induce the appropriate light response (L_x_).

When a PS is equipped with some kind of intelligence, in addition to the sensing signal or instead of it, the sensing system can provide an actuation signal; the sensing device is then transformed into a Smart Photonic Sensor (SPS) [26,27]. Thus, an SPS can be understood as the photonic sensor system that includes intelligence capable of offering actuation signals to allow appropriate reactions or interventions on/in the object (O_y_) from which the L_x_ modulated light is coming. This intelligence, commonly located in the optoelectronic unit, is created by software that executes specific algorithms.

According to the main technology used to build these sensing devices (mainly in the transducer part), several types or subdivisions of PS can be identified: Fibre Optic, Integrated Optic, Image, Hybrid. Sensors appear when Fibre Optic, Integrated Optic, Image-based or hybrid photonic technologies are used, respectively [28,29,30].

Overall, a Biomedical Photonic Sensor, BPS, is the photonic sensor to perform detection or/and measurement of biomedical measurands on/in a given object, providing as a result, representative electrical signals of its (object) state [30]. By using biomedical photonic sensors, measurands of a physical state (such as Body Temperature, Blood Pressure, Blood Flow, Heart Rate, Force, Position, Respiration, Shape Sensing, etc.), of a chemical state (pH, pO_2_, pCO_2_, Oximetry –SaO_2_, SvO_2_–, Glucose, Bile, Lipids, etc.), of a biological state (Antigens, Antibodies, Electrolytes, Enzymes, Inhibitors, Metabolites, Proteins, etc.), among others, could be detected or/and measured [31,32,33].

This work focuses on recent advances in biomedical photonic sensors in which optical fibres play a central role, i.e., biomedical optical-fibre sensors (BOFS). For instance, besides serving as light guiding media, the optical fibres act as transducers detecting signals from the measurand and enabling the characterization and calibration measurements of biomedical parameters.

## 3. Advances in Optical-Fibre-Based Biomedical Photonic Sensors

### 3.1. Physical Parameters

This section groups the most recent advances in physical parameters into two main applications: monitoring of vital signs and temperature of tissues (i.e., during laser ablation). In general, for physical parameter sensing, fibre Bragg gratings and Fabry–Pérot interferometers (FPI) are the key technologies employed. Further information can be found elsewhere for other physical parameters and previous works [2,3,34,35,36,37].

#### 3.1.1. Temperature

Most recent works on temperature optical-fibre sensing in biomedical applications relate to spatially resolved temperature measurements (or temperature profiling). Most of these works are linked to tissue laser ablation. However, temperature profiling and mapping also have applications in other medical procedures such as intravascular flow measurement, hot-wire anemometry, radiofrequency, or photothermal spectroscopy, among others [38].

Morra et al. presented a benchmarking of fibre Bragg grating (FBG) quasi-distributed sensing and Rayleigh scattering distributed sensing for spatially resolved thermometry [39]. In principle, FBGs are preferable over distributed sensing because of their lower cost of interrogation units [40]. The measurements were performed in tissue phantoms and an ex vivo pancreas subjected to contactless and contact laser ablation (deep-in-tissue). One-, two-, and three-dimensional temperature maps were obtained with a network of approximately 1 mm-long FBGs in 10-25-40 arrays. A laser spot (no information of spot profile was given) of 10 mm-diameter was focused on the centre of the sensor’s arrays for contactless ablation, and a laser applicator was used for contact ablation (minimum distance from sensor was 2 mm). They found that the lower the spatial resolution, the higher the root mean square error (<2.1 °C for 10-FBGs array) in temperature measurement when compared with a reference. The three-dimensional measurement confirmed the thermal spatial gradient in the pancreas subjected to laser ablation when using a highly dense FBG array (420 FBGs with 1 mm-sensing length). About 28 °C over a 5 mm temperature gradient was registered and 7 °C/mm close to the laser tip after 1 min of ablation. Tissues can reach high temperatures (over 100 °C) and high thermal gradients may occur, as confirmed by previous results. Hence, these large differences highlight the necessity of high-resolution, spatially resolved measurements for laser ablation of tissues. In addition, the FBG sensors showed better signal-to-noise ratios over time and lower standard deviations for temperature measurement. Regarding FBG sensors, in another study, the temperature monitoring of ex vivo breast fibroadenoma tissue was also demonstrated, providing real-time feedback to clinicians [41].

**Figure 3:**
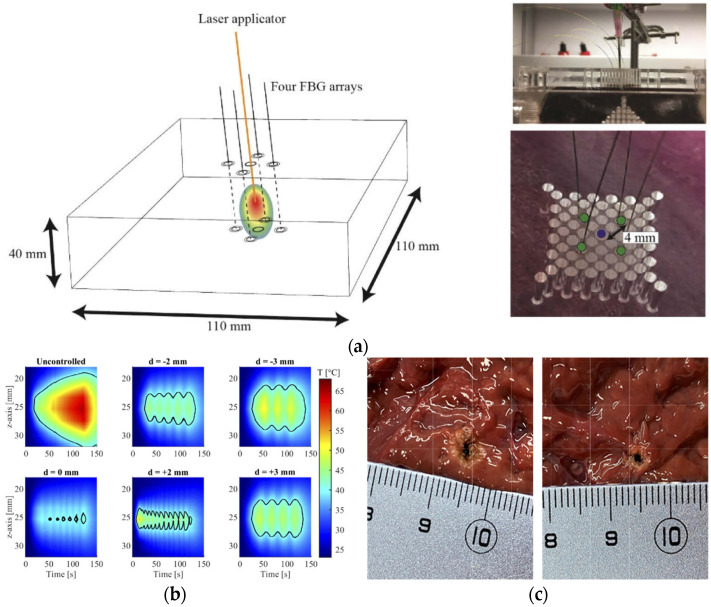
(**a**) Experimental setup: (**left**) schematics of the positioning of laser applicator and FBG arrays in the liver placed in a plexiglass box; (**top right**) photo of the experimental box employed for maintaining the position of the fibres; (**bottom right**) close-up of the box with labelled holes for the placement of the fibre-optic sensors (green colour, diameter of the hole equal to 1 mm) and the laser applicator (blue colour, diameter of the hole equal to 1.5 mm) inside the ex vivo liver. (**b**) Two-dimensional temperature map (time vs. distance along the controlled sensor positioned along the z-axis) for the uncontrolled ablation treatment and the temperature feedback-controlled ablations. Setpoint temperature is 40 °C, and *d* represents the distance between FBG array and the applicator laser tip. The black contour lines define the region of hepatic tissue at a temperature ≥ 40 °C. (**c**) RBG images of the thermal results for controlled (**left**) and uncontrolled (**right**) treatments at *d* = 0 mm. Images and caption adapted with permission from [42] © The Optical Society.

Several works have reported the quasi-distributed monitoring (spatially resolved) of temperature during laser ablation. Real-time monitoring of temperature for laser ablation regulation is not typically considered. In this context, Korganbayev et al. reported a closed-loop temperature control (spatially resolved) system for monitoring the temperature of ex vivo liver tissue during laser ablation [40]. It included highly dense FBG arrays that were inscribed in polyamide-coated single-mode-fibres. The device demonstrated control of the tissue temperature during contactless irradiation in the desired area. Nevertheless, this proof of concept was limited to a superficial treatment, and contactless irradiation is not an actual situation in clinical settings [42]. Hence, later, Orrico et al. moved towards a real application of laser ablation [42]. The control system comprising quasi-distributed FBG sensors (spatial resolution of 1.2 mm and thermal sensitivity of (7.43 ± 0.01) × 10^−6^ °C^−1^) was used interstitially (contact modality), including a simulation-based pre-plan model. The system allowed the margins of ablated zones to remain under critical temperature values (Figure 3). Finally, the strategy enabled the laser-induced temperature increase to be confined with higher accuracy in areas where sensors were located in the beam propagation direction [42]. In the context of laser ablation application, a different study reported the thermal properties of some ex vivo tissues (liver, brain, and pancreas) as a function of temperature [43].

A different strategy to FBG for 2D distributed temperature sensing was reported in [44]. Temperature monitoring was achieved by using a distributed sensor during nanoparticle (NP)-mediated ablation. The nanoparticles (20 nm-gold and magnetic iron oxide, which provide ease of synthesis, unique properties, and biocompatibility [44]) were deposited in the tissue assisting the laser ablation of a porcine liver phantom. The setup includes a fibre-coupled laser diode (980 nm), an optical backscatter reflectometer (OBR) and four sensing high-scattering nanoparticle-doped fibres. The fibres are doped with MgO [45] for enhanced backscattering. The sensing fibres enable the extended use of the OBR distributed sensing to multiple fibres and a 2D surface thermal measurement in real-time during 2D thermal ablation. The temperature changes are measured by analysing the backscattering profiles of nanoparticle-doped fibres, operated by multiplexing the signals and interrogated them using optical backscattered reflectometry. The spatial resolution is 3 mm, which is comparable to magnetic resonance imaging. Overall, the setup can be suitable for operation in vivo.

Finally, often, the dynamic response of sensors due to rapid temperature changes is not available. In 2020, Coote et al. implemented a Fabry–Perot (FP) cavity in a polymer-based optical fibre for the dynamic response of the temperature sensor [38]. They combined optical heating with pulsed laser and heat transfer models, suggesting the sensor’s suitability for monitoring temperature in thermal treatments. The sensor exhibited time constants on the order of milliseconds. They found the dynamic response was highly influenced by the medium (i.e., water or air) and deviation from first-order instrument response. This deviation was attributed to inhomogeneous temperature distribution in the medium, as suggested by the heat transfer model applied, with important implications for real applications.

#### 3.1.2. Vital-Sign Monitoring

Vital-sign monitoring includes the body’s essential functions such as pulse rate, body temperature, respiration rate, blood pressure, and others. Optical-fibre sensors can improve vital-sign monitoring of the human body [2]. Miniaturization and immunity to electromagnetic fields are essential characteristics of optical fibres that could enable their use, for instance, in magnetic resonance (MR) interventions and reduce the burden to patients, avoiding bulky measurement systems. Most common sensors are based on FBG, long-period fibre gratings (LPFG), and interferometers (e.g., FPI) [2]. Regarding fibre type, in general, silica and polymer optical fibres (POF) are commonly used. POF offers higher flexibility and lower cost compared to silica fibres. On the contrary, the body’s (organs, muscles) motion could influence the signals measured by the fibres. Therefore, motion should be considered, especially since POFs have a higher sensitivity to mechanical parameters due to their lower Young’s modulus than silica [46]. The latest works regarding pulse wave monitoring, respiratory rate, and blood pressure sensing are described below.

**Figure 4:**
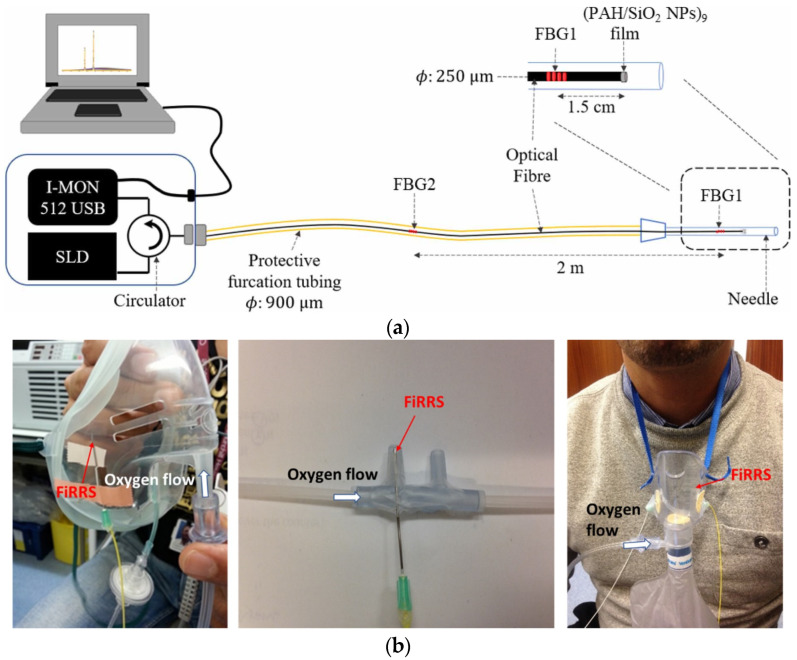
(**a**) Fibre-optic respiratory rate sensor (FiRRS) system, including a laser diode (SLD), spectrometer (I-MON 512USB), optical circulator, recording laptop and fibre-optic respiratory rate sensor enclosed within a 21-gauge hypodermic needle polished at the tip. (**b**) Integration of the sensing probe into (**left**) CO_2_ mask (with EtCO_2_ capnometry), (**middle**) nasal cannula and (**right**) non-rebreathe trauma mask. The image and caption were adapted from their original created by Sinha et al. [47] under a Creative Commons Attribution 4.0 Unsorted license.

Monitoring of respiratory rate is critical for the clinical care of patients, especially after surgery. Respiratory failure after surgical procedures can be significant (3.4%) in patients following an epidural and general and spinal anaesthetics [47]. In general, respiratory rate measurement is more challenging than that of heart rate, blood pressure, and oxygen saturation [47]. In this context, a novel system including FBGs to measure reflected signal for breathing and humidity sensing films (PAH/SiO_2_ NPs deposited at the tip of optical fibre) was developed by Sinha et al. in 2021 (Figure 4) [47]. The sensor was used in 15 healthy volunteers and differentiated between normal and low tidal volumes (*p*-value < 0.05). Such a study demonstrates that sensing reflectance techniques with optical fibres exhibit similar accuracy to capnometry and could be suitable for some oxygen delivery devices, i.e., for portable and continuous monitoring of respiratory rate.

Haseda et al. presented blood pressure measurement and pulse wave signals implementing a FBG sensor in POF. They attached the sensors directly onto the skin at the level of the brachial artery of the left elbow. The pulse wave for blood pressure was up to eight times higher than with silica optical fibre. They also found that measurements should be made on a wide range of reference blood pressures to confirm the reliability of the approach [48].

Laser speckle techniques can be used to measure physical (and chemical) parameters. It is a simple and powerful method for 2D mapping of flow and vibration [16]. Indeed, extensive research has been performed on monitoring microvascular blood flow utilizing speckle imaging techniques [49,50]. Regarding optical fibres and laser-speckle techniques, Rodríguez-Cuevas et al. presented a noncontact, continuous, patient monitoring using fibre speckelgram sensors based on polymer optical fibres [51]. The sensors were conceived to measure the heart rate and motion activity of patients lying in bed as a simple, robust, and low-cost alternative. Measurements of movement and heart rate in 20 volunteers were performed employing two sensors located in distinct parts of the bed. Different data processing methods were evaluated, and the optimal processing method exhibited high accuracies (below 3% instantaneous error, comparable to other optical-fibre sensors [2]) and heart rate mean difference with the reference below 10% beats per minute [51]. More recently, a comparison of postprocessing data methods for breath and heart-rate sensing was performed on ten volunteers [52].

A strong potential niche for optical-fibre sensing is their use during MR interventions. For example, a FBG breathing sensor was recently reported for application in actual practice [53]. The sensor is simple, low-cost, and fully MR-compatible. The sensor was tested in the laboratory using real MR, exhibiting a relative error < 5%. It can serve as respiratory triggering and can monitor the development of respiratory rate in the MR environment; and can be helpful in preventing hyperventilation syndrome.

Finally, Ushakov et al. compared several interferometric fibre-optics sensing techniques for pulse wave monitoring, including FPI, FBG, optical coherence tomography (OCT) and a singlemode-multimode-singlemode interferometer [54]. The benchmark was based on a series of quality metrics from the pulse wave signal. These metrics were focused on signal-to-noise ratio, robustness, and repeatability of measurements against motion artefacts when monitoring pulse wave of different arteries (carotid, subclavian, and radial arteries). OCT resulted in the most robust approach against motion artefacts, providing the best-demodulated signal quality. However, its widespread implementation for routine measurements is less realistic. For instance, FPI sensors are more suitable for portable devices, despite the relatively lower performance demonstrated.

### 3.2. Biochemical Parameters and Biosensors

This section presents the sensing of biochemical parameters by several techniques, including biosensors. Optical-fibre sensing of biochemical parameters requires direct interaction with the measurand. Commonly, tapered fibres, diffraction gratings (at the end-face of the fibre), FBGs (at the end of the fibre and/or within the tapered region), and LPGs (excitation of cladding modes) have been traditionally used. Then, surface plasmon resonance effects, enabled by metal coating (e.g., Au or Ag) of the fibres in different configurations (e.g., U-shape, tapered, on or at the end-face of the fibre), accomplished an increased sensitivity of many parameter’s measurements by several orders of magnitude. More details on the use of plasmonics in the field of sensing can be found in [55].

The use of biosensors has added another degree of sensing capability. Optical fibres using a biorecognition element (antibodies, antigens, proteins, enzymes, nucleic acids, antigens, or cells in general) are considered optical-fibre biosensors [56,57]. These biological elements could recognize biochemical measurands, for instance, cancer cells. The biorecognition elements are bound to the fibre via covalent bonding. In other cases, they can be physically trapped in hydrogels [57]. The sensing can be performed in label or label-free mode. Alternatively to SPR, noble metal nanoparticles enable modified versions of SPR sensing [57]. The most common is based on localized surface plasmon resonance effect (LSPR). Then, new possibilities for sensing appeared, for example, Surface-Enhanced Raman Scattering (SERS) [57], including optical-fibre SERS sensing [58,59,60,61,62].

Examples of BOFS for pH and oxygen detection can be found in Section 3.2.1 and Section 3.2.2, respectively, whereas those for cancer detection and diagnosis are presented in Section 3.2.3. In addition, the detection of other diverse biochemical parameters has been recently reported and will be detailed in Section 3.2.4 [63,64,65,66,67,68,69,70,71,72].

#### 3.2.1. pH

Variations in pH can be caused by, or due to, a disease or misfunctioning of parts of the human body. Thus, pH is a quantity of paramount importance for sensing in biological systems. Among others, pH determination from ex vivo (e.g., urine, saliva) and in vivo (e.g., intra and extracellular pH) is desirable. Furthermore, pH measurements could also assist in the discrimination of normal and tumour cells. Commercial pH sensors measure the potential difference between two glass electrodes with a detection range of 0–14 and a resolution of 0.01 pH [73], and about 0.003 pH in a more restricted range (5–7 or 6–8 pH) [74]. The most common methods for pH measurement using optical fibres (monitoring fluorescence, absorption, lifetime) employ dye indicators or fluorophores deposited on the tip of the fibre [75] or in U-shaped [73] or tapered optical fibres [76]. Typically, such sensors combine fluorescence principles with a variation of the evanescent field by tapering the fibres [75]. Besides the general benefits from optical-fibre sensors (miniaturization and immunity to electromagnetic fields), they exhibit high sensitivity. On the contrary, one challenge is that dye-leaching and photobleaching reduce the long-term stability and sensitivity [77].

In 2018, an outstanding optical-fibre sensor using a dual excitation ratiometric detection method was reported by Wencel et al. [78]. The fibre-based fluorescent sol–gel-derived sensor uses a multicore fibre and targets real-time and continuous monitoring of in vivo pH in human tissue. Before in vivo measurements, the system has been extensively tested in the laboratory (e.g., long-term stability, temperature and ionic strength cross-sensitivity and biocompatibility). A stable hybrid sol–gel pH-sensitive material is deposited on the tip of plastic fibre. The fibre sensor is insensitive to bending (critical for in vivo applications), exhibits a resolution of 0.0013 pH units (outperforming state-of-the-art pH sensors), and drift of 0.003 pH units per 22 h in a laboratory environment (0.004/h in vivo) [78]. The maximum sensitivity was accomplished in the physiological pH range with a dynamic range between 6 to 8 pH units (response time, *t*_90_ < 2 min).

**Figure 5:**
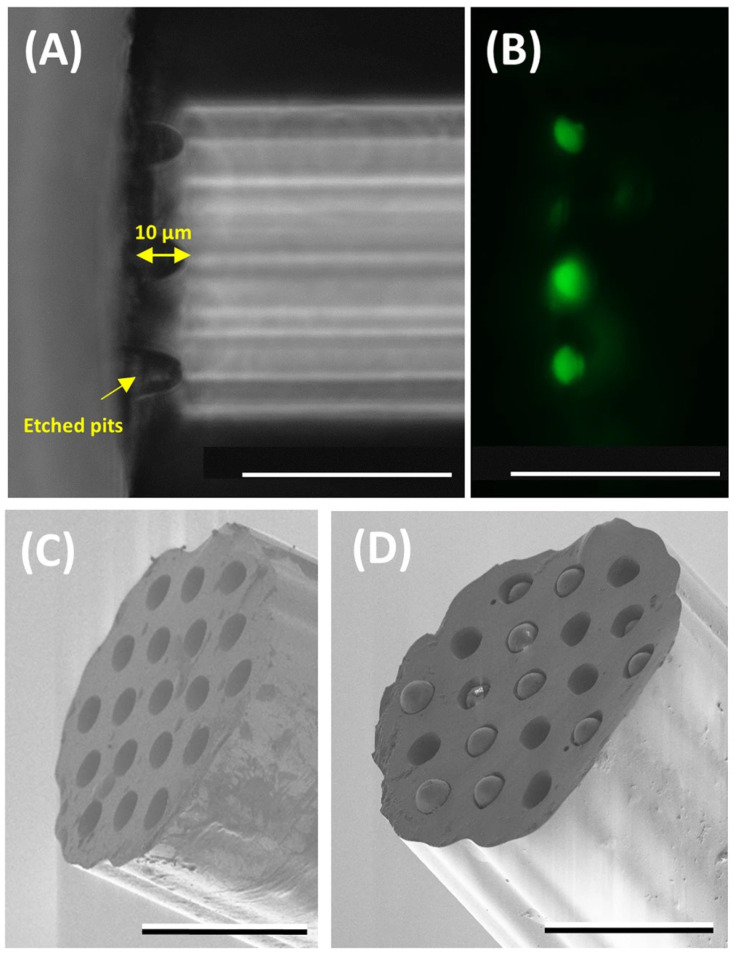
Optrode fabrication and characterisation. (**A**) Bright-field image of a fibre (viewed from the side) with etched pits (~10 µm in depth). (**B**) Fluorescence image (excitation 488 nm and emission 520 nm) of the etched fibres (viewed from the side) after the pH sensors (fluorescein-based) were loaded into the pits. Note the focal plane of the image in (**B**) is different from (**A**) to highlight the loaded cores. (**C**,**D**) SEM images of an etched optical fibre before and after the addition of the microspheres. The scale bar in all the images is 50 µm. The images and caption were taken from their original created by Choudhary et al. [79] under a Creative Commons Attribution 4.0 Unsorted license.

In 2019, a flexible miniaturized optrode was reported using multicore optical fibres and novel chemical probes to sense pH and oxygen [79]. The sensing technique was conceived to perform measurements deep in the tissue (i.e., ex vivo lung model), which was enabled by the size of the multicore fibres. The fibre has a 200 µm diameter with 19 cores that could act as independent sensors. Silica microspheres are placed in the cores (10 µm-diameter) at the end of the fibre after etching (Figure 5). Then, fluorescein-based sensors were attached covalently to the microspheres for pH sensing and palladium porphyrin complex based for oxygen sensing. Both sensors detect the fluorescence emission changes in response to pH and oxygen, critical parameters for monitoring lung health. A high-fidelity measurement with high sensitivity was achieved by performing ratiometric sensing (resistant to laser power fluctuations). An accuracy of ±0.02 pH units and oxygen sensor accuracy of 0.6 mg/L were demonstrated. The architecture of the sensor is flexible and could allow for multiple sensors. Interestingly, a novel phenomenon was reported regarding the fluorescence behaviour of the pH sensors. A high-density deposition of the carboxyfluorescein showed an inverse shift in fluorescence in response to pH changes.

Functionalization of the optical fibres has brought higher sensitivity for the measurements; however, there is still a need to maximize the signal-to-noise ratio in some instances. The density of fluorophores at the tip of an optical fibre plays an essential role in the sensor’s sensitivity and the dynamics of the signal detected. This has been shown by Ehrlich et al. [80]. The authors pursued sensing parameters for in vivo applications using the same fibre sensors as explained previously [79]. They noticed that a high density of fluorophores at the tip of the fibre provides higher sensitivity and stability for measuring pH. In addition, they introduced time-resolved measurements of the fluorophores emission to provide insight into the dynamic changes occurring when the density of fluorophores was modified.

In 2020, a hydrogel-based optical-fibre fluorescent pH sensor for observing lung tumour tissue acidity [81] was reported with a precision of 0.1 pH units (dynamic range from 5.5 to 8.0). The sensor was fabricated by in situ photopolymerization processes and validated against a commercial glass-electrode pH meter. Measurements across a whole ovine lung were performed and the sensor discriminated between normal and tumour tissue. Real-time rapid observation of pH changes (response time of 30 s) could be measured, offering the potential for sensing platforms with continuous monitoring of multiple parameters.

In 2021, Podrazký et al. reported pH measurement by a tapered optical fibre with dye immobilized at the tip by a hybrid sol–gel matrix at the tip of the fibre [76]. The sensor was tested in cataract surgery in two groups of patients and exhibited a precision of the measurement ±0.04 pH units. Furthermore, a dye-free (avoiding dye-leaching and photobleaching) fibre-optic pH sensor was proposed in the same year. The optical fibre is U-shaped with an pH-sensitive membrane embedded by hydrogen bonding ethylcellulose with a sol–gel matrix [73]. The sensor exhibited reversible response within 4.5 to 12.5 pH units with low-temperature cross-sensitivity and accuracy of ±0.2 pH units. Finally, for biomedical interest, the sensor was tested to monitor the pH of human serum.

#### 3.2.2. Oxygen

Luo et al. reported a new method for sensing dissolved oxygen with good sensitivity and linearity [82]. Commonly, optical-fibre sensing of dissolved oxygen is achieved by the fluorescence quenching effect of oxygen molecules with embedded fluorescent molecules in a gel or polymer matrix [83]. Alternatively, Luo et al. sensor is based on SPR (gold-coated fibre) and the oxygen transport mechanism through haemoglobin (modified on the surface of the gold film). In the presence of oxygen, the haemoglobin is converted to oxyhaemoglobin resulting in a change in refractive index, traceable by SPR sensor. The linearity and sensitivity were improved using carbon nanotubes. The detection sensitivity is 8.89 nm/(mg/L) in the dissolved oxygen range of 2 to 20 mg/L, about 2.8-fold higher than without carbon nanotubes. The limit of detecion is 0.01 mg/L. The sensor has good repeatability and stability without consuming oxygen during the process. It is worth mentioning that fluorescence-based sensors do not consume oxygen either. Regarding oxygen measurement with biosensors, another study presented promising results tackling the challenge of nonreversible sensing when using biosensors. A reversible oxidative stress sensor was demonstrated [84]

Recently, Fru et al. designed a novel interstitial diffuse optical probe to perform near-simultaneous measurement of blood fraction and oxygen saturation in tissue [85]. The optical probe was used to guide the pump light and collect diffuse light from tumour-bearing mice. They follow the Jacques et al. method to retrieve the biological composition of the tissue from optical measurements [85,86]. The results obtained were benchmarked with photoacoustic imaging, achieving a positive correlation between blood volume and oxygen saturation. Such a strategy is an excellent example of the useful application of optical fibres. Besides monitoring measurands, it could provide information for a better understanding of the dynamic behaviour of tumour hypoxia in cancer treatments, i.e., photodynamic therapy. A descriptive comparison with other measurement systems is included.

#### 3.2.3. Cancer-Related

Typically, for cancer diagnosis, the workhorses are standard flow cytometry analysis and Raman spectroscopy regarding optical techniques. On the other hand, most widespread optical-fibre sensors for cancer detection/diagnosis are also based on SPR sensing techniques and biosensors. Regarding the latter, they are widely used for cancer detection [87,88].

Towards in vivo sensing, the detection of specific cancer biomarkers using an innovative biosensor was reported by Ribaut et al. [89]. The sensor is based on SPR and includes a tilted FBG to detect lung cancer biomarkers in situ (cytokeratin 17). The sensor was encapsulated in a gel matrix (to mimic tissue samples) and inserted into human lung tissue (ex vivo). A stable signal was achieved, and the resulting spectra confirmed good selectivity and specificity response and high sensitivity (1.5 dB at 1 × 10^−10^ g/mL shift of the most sensitive mode) of the biosensor.

The use of optical-fibre aptasensors can also be found in recent literature. The so-called optical aptasensor uses aptamers as biorecognition elements in biosensors. Aptamers bind to and identify different cancer biomarkers (or selected targets) with high affinity and specificity. Loyez et al. demonstrated the rapid detection of circulating breast cancer cells (in vitro) at very low concentrations using an optical-fibre aptamer-based strategy [90]. This detection is challenging in circulating tumour cells because they exhibit low concentrations, typically 1–10 cells/mL of blood. Hence, high sensitivity is required. A plasmonic tilted FBG (pTFBG) sensor included a previously selected aptamer (MAMA2). Detection of 100 cell/mL was achieved with a label-free detection strategy (limit of detection 49 cells/mL). Detection of 10 cell/mL was achieved using gold nanoparticles as a signal amplification tool. Accordingly, this work’s sensors and detection sensitivity have strong potential for their use in cancer detection, especially for circulating tumour cells responsible for metastasis. Further information regarding optical aptasensors for cancer can be found elsewhere [91].

**Figure 6:**
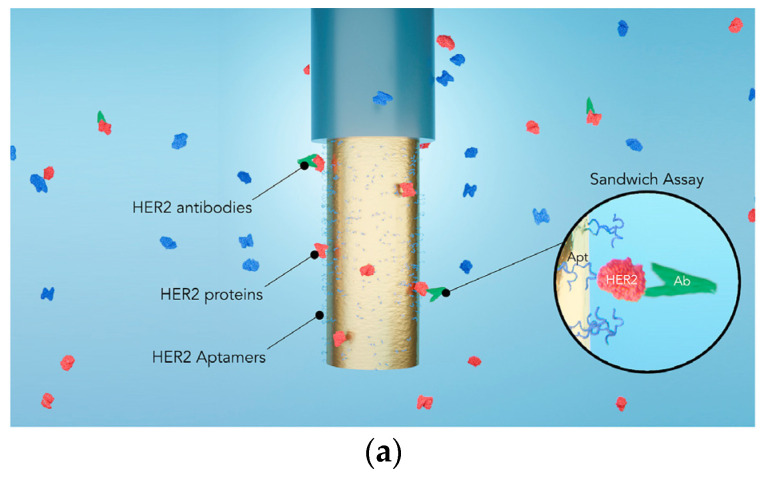

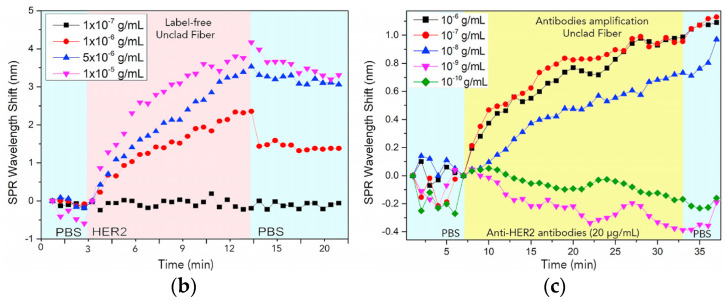
(**a**) Sketch of the gold-coated fibre used to detect HER2 molecules (in red) through SPR, with antibody amplification in a sandwich configuration (in green). Thiolated aptamers are immobilized on the gold surface to target HER2. (**b**) SPR-dip minimum shift as a function of time in phosphate buffer saline (PBS), HER2 proteins, and PBS after the immersion in HER2 solution. (**c**) SPR-dip minimum shift as a function of time in PBS, in antibodies (anti-HER2), and in PBS after amplification. Each curve represents one probe per test. Reprinted (adapted) with permission from [92].

Later, in 2021, SPR optrodes were reported for HER2 breast cancer biomarker detection [92]. The optical-fibre sensor uses a step-index multimode optical fibre coated with gold (film thickness influence was studied). Then, an optical-fibre-based sandwich assay was performed implementing HER2 aptamers/HER2 proteins/HER2 antibodies for cancer biomarker detection (Figure 6a). Different performance indicators analysed the sensitivity of the optrode after processing the central wavelength of resonance shift and the refractive index (Figure 6b,c). The optrode targets real-time monitoring, and multiple assays confirmed the results. For example, HER2 biomarkers had a sensitivity of 1 μg/mL (~8 nM) in label-free mode, whereas the amplification with HER2-antibodies provided a nearly hundredfold signal magnification with 10 ng/mL (~86 pM) sensitivity.

Other new developments have been carried out towards cost-effective solutions and miniaturization of the sensing systems [93]. For example, Etcheverry et al. reported a high-performance flow cytometer based on optical fibres [93]. The all-silica fibre microflow cytometer measures fluorescence and scattering from particles and cells. Light delivery can be accomplished through optical fibres and cell (colon cancer cells) transport in circular capillaries. High flow rates (up to 800 µL/min) were demonstrated, enabling a throughput of 2500 particles/s. The authors state this system (robust, portable, and low-cost) could be the basis for a point-of-care flow cytometer with a comparable performance to commercial systems.

#### 3.2.4. Other Biochemical Parameters

Immunosensors and mainly paper-based dip sticks perform detection of PfHRP2 [63]. The paper-based method provides cost-effective and straightforward detection. However, sensitivity to HRP2 is not the highest and could lead to false-negative results. In this context, Loyez et al. presented an optical-fibre sensor based on SPR using a thin, gold-coated layer on fibre surface [63]. The sensitivity achieved was 1 µg/mL (projected improvement by a factor of 10). The current lowest sensitivity reported to date is 0.8 ng/mL or about 200 parasites/µL. Although the sensitivity is not the highest, the strategy used a multiplexing system in which beneficial detection of different proteins could be achieved. For instance, it seems there could be a preference on probing LDH over HRP2, and in this case, multiplexing could allow the detection of both, providing additional information. As mentioned previously, one crucial challenge for optical biosensors is that their response is nonreversible. To tackle this challenge, a reversible in vivo biosensor based on optical fibres was developed to measure protein carbonylation [69]. Other recent results are related to SPR optical-fibre sensors for immunoassays, including nanosheets, disposable, and optofluidic systems (for p53 protein) [70,71,72].

Recently, a plasmonic tilted FBG grating sensor (see [94]) based on a p-mercaptophenylboronic acid monolayer and Au nanoparticles for highly sensitive and stable glucose detection was reported [65]. Blood glucose concentration in an average person ranges from 80 to 120 mg/dL (4.4 to 6.6 mM) [66]. In this work, a concentration of glucose in the pM level with a competitive limit of detection of 295 pM (maximum sensitivity of 1.08 dB/nM) was accomplished. The sensor exhibits a very large dynamic range from 1 nM to 10 mM. Good selectivity of fructose and galactose was reported. The amplitude demodulation method was employed, offering minimal temperature cross-sensitivity. The sensor offers capabilities for non-invasive point-of-care testing and tumour microenvironment research. Another example on the use of plasmonic tilted FBG sensors was used for calmodulin detection [64]. The sensor includes a tilted FBG by 18 °C and a 50 nm-thick gold nanofilm coating on the fibre surface. The sensor achieves a limit of detection of 0.44 nM. Combined with a microfluidic system, the sensor can be used for biomolecule detection and interaction monitoring in real applications.

In 2021, an SPR fibre sensor with gold nanoparticles (40-nm diameter) for measuring Cytochrome c (Cyt c) was reported by Ortega-Gómez et al. [67]. Typically, flow cytometers, Raman spectroscopy, or time-consuming methods such as ELISA or Western blot are used to detect Cyt c. In this study, a new approach using optical-fibre sensing was demonstrated. Instead of focusing on the shift of the plasmon resonance peak, they monitored the absorption changes of the plasmon resonance spectrum. As a result, a concentration of up to 80 µM and limit of detection of 60 nM was achieved. These properties are comparable to other standard methods (electrochemical). Furthermore, Cytochrome c plays a major role in cell apoptosis; thus, the sensor can be used in cancer treatments/identification, as a cardiovascular disease marker, and in early diagnosis of other diseases.

Finally, an optical-fibre aptasensor (ELISA-like sensor) using an in-line microcavitity MZI was developed by Janik et al. using fs-laser processing [68]. The sensor used peptide aptamers as bioreceptors to measure *E. coli* O157:H7. The authors achieved one of the most sensitive and lowest-analyte-volume label-free optical-fibre biosensors. The lowest detected volume was 10 CFU/mL, comparable to other detection methods. The sensor reduces false-positive measurements.

Table 1 summarizes the details and main findings of the works described in this section, including some remarks regarding reference literature.

### 3.3. Multiparameter Sensing and Lab-on-Fibre

This section includes results on multiparameter sensors and lab-on-fibre or lab-in-a-fibre devices. In the following, lab-on-fibre or lab-in-a-fibre will be referred to as LOF. A brief introduction to such concepts is given before describing the latest results on multiparameter sensors (simpler structures) and more advanced optical-fibre sensing structures towards LOF devices.

**Figure 7:**
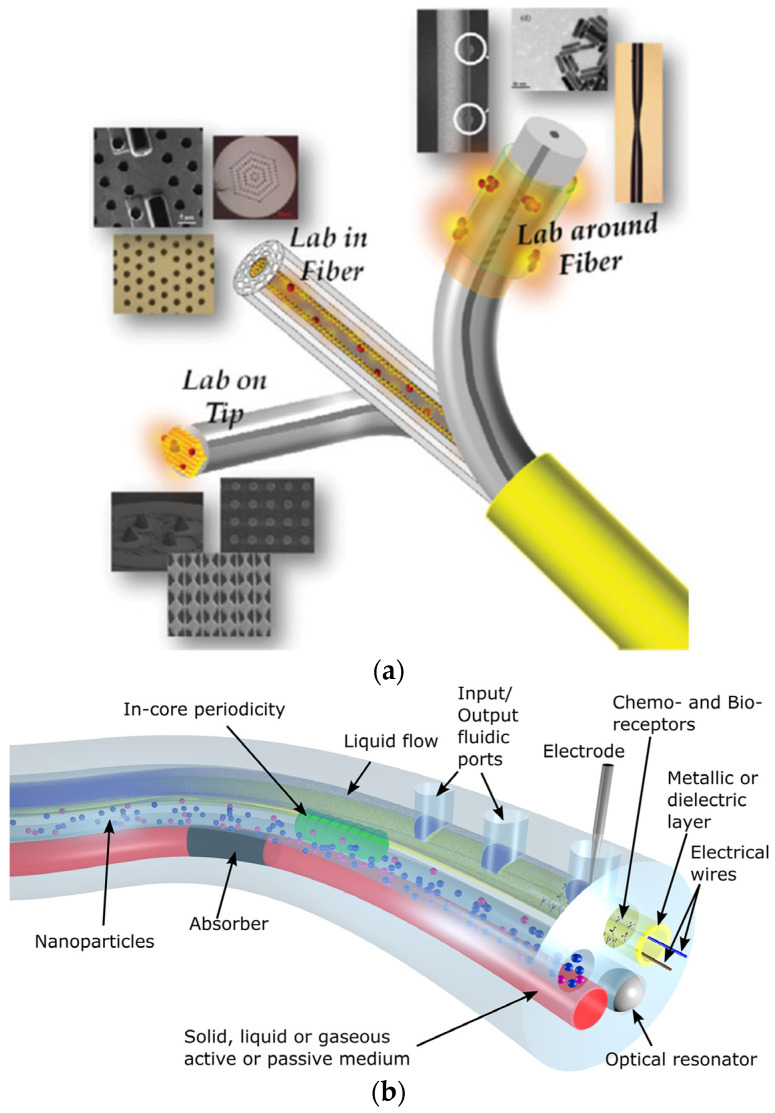
Illustrations of lab-on-fibre approaches. (**a**) Reprinted (adapted) with permission from [97]; (**b**) Reprinted (adapted) with permission from [95].

LOF technology has become an important research area towards miniaturization and cost-reduction of systems with outstanding features and strong potential for biomedical applications. Lab-on-fibre can be conceived as a platform including multifunction sensing and/or actuating systems (Figure 7) [95,96]. Another possibility integrates optical elements (e.g., microlenses) into functionalized fibre tips whose combination provides multiple functionalities. Moreover, the combination of optical fibres with microfluidics [10] (optofluidics) and optomechanics [96] is also enabled by LOF technology. For a side-by-side comparison between LOF technology and lab-on-chip, the reader is referred to [95].

Lab-on-fibre technology with different capabilities can be accomplished by using several fabrication techniques. For instance, nanolithography, self-assembly processes, FIB milling, electron beam lithography, two-photon lithography (or two-photon polymerization), and 3D printing. Many of these techniques have been described by previous extensive reviews of LOF technology [95,96,97].

Alternatively, lab-in-a-fibre devices can also be fabricated by femtosecond laser irradiation followed by chemical etching (FLICE) [11]. FLICE, also referred to as ultrafast laser-assisted etching (ULAE) or femtosecond laser-assisted etching, has unique capabilities that allow straightforward fabrication of channels (with natural circular cross-sections, not easily achieved with other techniques) with 3D geometries for sensing [11]. Besides typical inscriptions using femtosecond laser processing (see [98]), the combination of fs-laser processing and chemical etching on silica (e.g., HF or KOH) has demonstrated the fabrication of microchannels, X-couplers, filaments, among others [11,99]. Inscribed microchannels could allow liquids to flow in and out of the fibre, expanding the ability to measure diverse parameters. For instance, microfluidics plus photonics (known as optofluidics [10]) could also be implemented in the fibre (making use of the cladding). Typically, FLICE is performed on silica fibres, whereas the inscription of microfluidic channels on polymer fibres has also been reported [100]. Although complex in its fabrication process, this new approach could expand the applications of optical fibres and photonic devices. In this context, the lab-on-fibre technology is no longer only restricted to transducing elements in, on, and around the fibre. Thus, the potential for biomedical applications, including multiple transducers in a single fibre, more functional surgical tools, medical probes or catheters, distributed sensing and microfluidics flow cytometry, can be envisaged. Another biomedical potential is the fabrication of shape sensing systems. For example, the measurement of torsion in a single fibre (typically requiring multiple fibres, see Section 4.2) was enabled by using FLICE to inscribe a twisted structure inside the fibre [101].

Regarding the applications of multiparameter sensors (simpler structures than LOF devices), just a few examples of multisensors have been reported for the biomedical field (some previously described in Section 3). On the other hand, there are other general studies on optical sensors for multiple parameters with strong potential for biomedical applications. In the following, we highlight some examples of optical-fibre multiparameter sensors targeting biomedical applications. Then, a couple of examples, including a lab-on-fibre strategy, will be described.

The simultaneous measurement of temperature and pressure is the most common example found in the literature [102,103,104]. A proof of concept for an intravascular temperature and pressure sensor was reported by Poduval et al. [104]. In addition, a temperature and strain sensor using a multi-interferometric serial configuration were recently reported [105]. Potential biomedical applications are then found in sensors with no cross-sensitivity between refractive index and strain measurements, as reported by Roldán-Varona et al. [106]. The sensor can be incorporated into a surgical needle for characterization of parameters such as the insertion distance and tissue optical properties [58]. Characterization of curvature and strain [107], refractive index, and temperature have been also demonstrated [108,109]. A strategy for automated capillary refill time and contact pressure measurement has been reported recently (in 2020) [110]. Authors state that capillary refill measurements are typically performed manually. Other examples of multiparameter sensing include ammonia and CO_2_ [111], temperature, and absolute humidity [112]. For a generic review on multiparameter optical-fibre sensors (not directly focused on biomedical applications), the reader is referred to [113].

Recently, first attempts towards the use of lab-on-fibre technology in biomedical applications have been carried out. For example, Managò et al. developed SERS optrodes by patterning three substrates covered with a thin gold layer towards a LOF device [58,114]. Nanolithography and self-assembly processes realized the nanostructures. The patterning was optimised for different biological targets, namely, blood cells and bovine serum albumin. They found that the sensor’s sensitivity depends strongly on the size of the target. Hence, the sensor design and patterning of the structures should be dependent on the biological target.

Paiva et al. developed a platform capable of identifying single-cell types for human cancer [115]. The motivation behind this development is that optical-fibre tweezers, which simultaneously sense and trap, are scarce, and immunoassays are time-consuming. Regardless, as mentioned previously, the workhorse technologies for cancer detection are flow cytometry (which is rather bulky and expensive) and Raman spectroscopy. Plasmonic resonance may be an option. However, it requires a complex multistep fabrication process, and higher refractive index sensitivity affects trapping capability. Hence, Paival et al. implemented a microlens into optical-fibre tip fabrication by photopolymerization. The device can be conceived as an optical tweezer, capable of trapping single cells and identifying them upon analysing laser back-scattered signals through the optical fibre. They achieved detection of cancer cells with different glycan content (a substance linked to tumours). This probe can immobilize the targets, identify single cells in human cancer, and distinguish very similar cells only differing by surface glycosylation.

## 4. Fibre-Optical Probes for Needles, Catheters, and Endoscopy

Optical probes based on fibre optics has been employed for many years in biomedical applications. Fibre optics have been used as a flexible solution to interface the spectroscope and the sample. In this case, the application usually measures a turbid medium (e.g., tissue), using amplitude, polarised reflectance, Raman, and fluorescence techniques. Besides using fibre-optical probes for interfacing, advances in micro- and nano-fabrication have enhanced probes based on gratings. In addition, several designs have been demonstrated inside needles and catheters for force and shape sensing. Another important class of fibres for shape sensing are those based on interferometric effects (Fabry–Perot).

This section is divided into two parts. First, recent advances in fibre-optical probes for spectroscopy are described, focusing on applications in turbid media, such as tissue. After that, recent advances in modified optical-fibre probes used in needles and catheters for biomedical applications are included. The searching of recent publications was restricted to the last five years.

### 4.1. Fibre-Optical Probes for Spectroscopy

Optical techniques have traditionally been performed in clinical procedures for decades [116,117,118]. From a medical point of view, the different existing optical biopsy techniques offer a detailed tissue fingerprint based on its chemical content [117,119]. Consequently, it provides clinically relevant information for detecting normal and cancerous tissue, treatment choice, or its status, among others [120,121]. Furthermore, within these optical biopsy techniques, it is possible to highlight fluorescence spectroscopy, fundamentally from optical endomicroscopy (OEM), as well as reflectance spectroscopy, with particular attention to Raman probes. These techniques can be incorporated into optical-fibre-based probes, as they are a genuinely suitable tool for clinical procedures [120,122]. Furthermore, they are flexible, handy, allow in vivo in situ tissue interrogation, and are minimally invasive, i.e., they will enable the integration of smart fibre-based devices in catheters with an external diameter of less than 1 mm. Finally, some of the most significant advances made in recent years are presented.

#### 4.1.1. Reflectance Probes

Among the optical-fibre probes related to spectroscopic techniques, those detecting the multiple-scattered light that escapes from the sampling volume are of paramount consideration. These are the reflectance probes [117,120]. Typically, in biological tissue, scattered light depends on the structural (which in turn depends on the size and shape distribution of the scattering particles) and chemical (tissue type, metabolic state, blood supply, among others) composition [123,124]. As all these optical properties vary spatially and with wavelength, and many studies have employed tissue-inherent diffuse reflectance spectroscopy to distinguish normal and abnormal tissue in vivo [125,126].

Typically, reflectance-based fibre-optic probes are based on a single fibre containing the excitation and several spatially distributed collector fibres to capture the reflected signal [127]. This signal is then processed, and the data is fitted to analytical expressions (diffusion theory) or iterative algorithms (Monte Carlo simulations) [120]. Although many techniques are based on diffuse tissue reflectance (capturing amplitude, polarisation, phase), those based on Raman scattering stand out above all others regarding the analysis of chemical species. The most relevant fibre probes based on these techniques in recent years are presented below. The selection of works includes only those using femtosecond laser processing for the manufacturing of the fibre sensors.

Within reflectance, Raman scattering detection is a valuable tool to perform histochemical analysis in a label-free, non-invasive, and non-destructive way [121,127,128]. However, one of the challenges of Raman-based probes is that the signal-to-noise ratio is weak [129]. Only $\sim 10^{-10}$ of the incident light is Raman scattered, and additionally it is $\sim 10^{6}$ weaker than fluorescence signals [120]. Furthermore, the laser source itself, the optical fibre, and other elements, introduce unwanted background signals. A background Raman signal is generated when a narrow pump (i.e., laser) passes through a silica fibre. This signal is large enough to overwhelm any signal collected from the biological sample [128,130]. Hence, the background Raman signal and the reflected pump signal must be prevented from being collected. Typically, notch filters prevent undesired signals from the laser, whereas the Raman background signal is often avoided using different fibres for collection and excitation. Correct design of the optical-fibre probes is needed to maximise the efficiency in capturing the desired signal [120,127,129].

**Figure 8:**
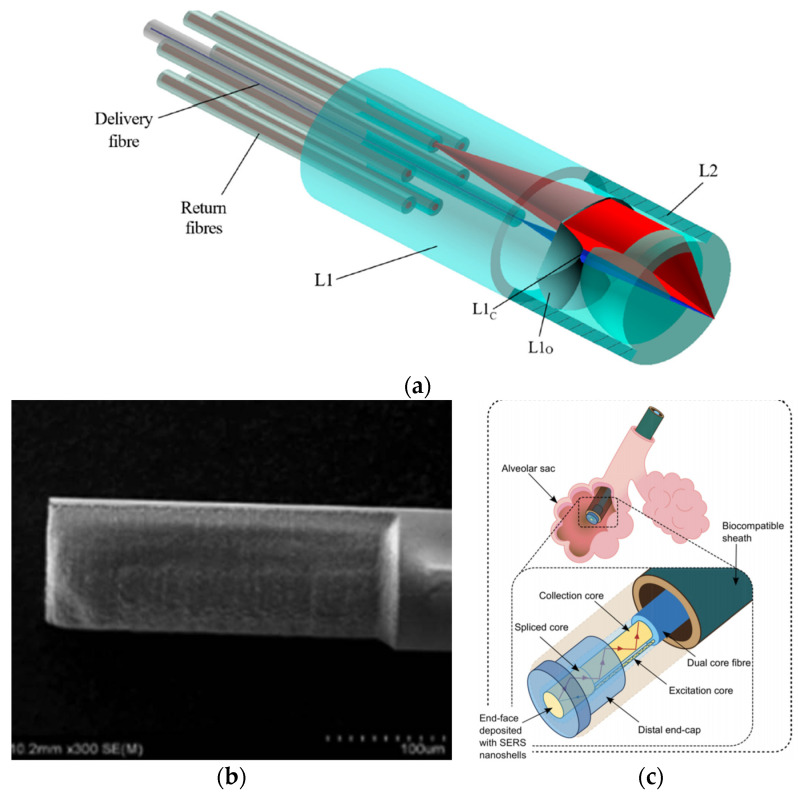
(**a**) Ultrafast laser-written Raman probe with one delivery optical fibre and six collector optical fibres. The fibre endcap (made up of two pieces) has been made utilising a combination of femtosecond laser-writing and chemical etching (image adapted from [129]). (**b**) Fs laser-ablated fibre based on a D-shape for enhanced SERS probes (adapted with permission from [131] © The Optical Society). (**c**) Probe for alveolar pH sensing. It contains a dual-core optical fibre for spatially separating the pump delivery and signal collection, gold nanoshells deposited on the end-face for SERS, and a fused silica endcap to provide robustness (adapted with permission from [130] © The Optical Society).

In 2020, Ross et al. developed a novel optical-fibre probe for Raman spectroscopy-based optical biopsy based on a micro-optic system for efficient signal delivery and collection (Figure 8a) [129]. The micro-optical system was manufactured by combining femtosecond laser writing and KOH chemical etching (final roughness of 2 nm). With a diameter of 960 µm, the probe has a collection efficiency of 71% over a 0.8 numerical aperture. In addition, measurements were performed on mouse intestinal tissue, verifying the probe results with a commercial Raman microscope.

In other studies, the femtosecond laser is only used to ablate the fibre and deposit nanoparticles, generating surface-enhanced Raman scattering (SERS) probes [119]. In a 2016 study, a D-shaped fibre probe was made with a femtosecond laser, subsequently depositing silver nanoparticles (Figure 8b) [131]. The results showed that the longer the D-shaped length (fs laser zone ablated), the more significant the increase in the SERS signal.

On the other hand, in the same year, a femtosecond laser was used to inscribe a 5 µm period surface grating on the end-face of a 130 µm diameter PMMA optical fibre [59]. After a photo-reduced deposition of silver nanoparticles, it was shown that the SERS signal is increased by around four times concerning the non-laser-processed structure. Due to the small size of the optical-fibre probes, it is possible to use them to analyse or treat tissues that are difficult to access, such as alveolar sags (Figure 8c). Choudhury et. al. developed an endoscope optrode based on an asymmetric dual-core optical fibre on the distal end face in which gold nanoshells (to perform SERS) have been deposited [130]. As a result, a ~100-fold improvement in signal to background (delivery and collection light paths are separated) is achieved. Hence, the measurements are no longer limited by the undesired Raman contribution from the fibre. Additionally, a fused silica endcap was manufactured by femtosecond laser inscription and selective chemical etching to provide robustness to the probe. Its performance was demonstrated for multisite pH detection with measurements in the respiratory acini of an ex vivo ovine lung model. The accuracy of the measurements was ±0.07 pH units.

#### 4.1.2. Fluorescence Probes

In this case, the latest developments in this field employ technologies other than femtosecond lasers. They are mostly related to coherent fibre bundles (CFB), also known as imaging fibres [120,132,133]. It is known that different human tissues contain chromophores, whose fluorescence intensity and spectrum shape are strongly dependent on the characteristics of the excitation light source [120]. Numerous clinical studies have also shown that fluorescence spectroscopy allows the determination of an abnormal state of human tissues in the brain, head, breast, skin, or gastrointestinal tract, among others [117,125].

**Figure 9:**
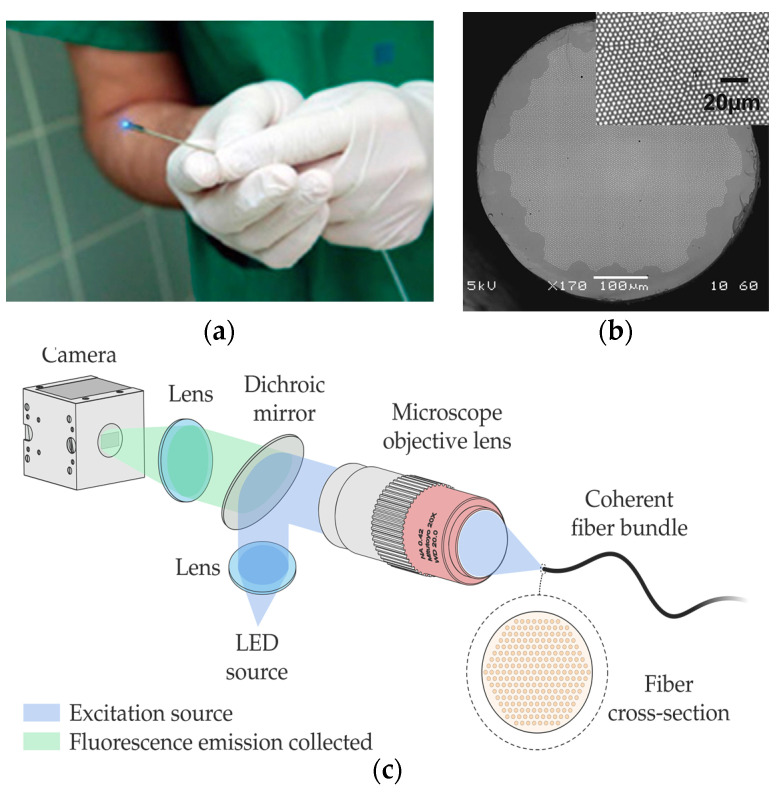
(**a**) Blue light-emitting optical-fibre probe for fluorescence microscopy in endoscopically accessible tissues (This image was taken from their original created by Osman et al. [133] under the terms of the Creative Commons Attribution 4.0 International License). (**b**) High-resolution hexagonal array imaging fibre comprises 12,247 cores. This image was taken from their original created by Stone et al. [137] under a Creative Commons Attribution 4.0 Unsorted license. (**c**) Example of an epifluorescence imaging system (image courtesy of the authors). An excitation source is coupled via a dichroic mirror into the coherent fibre bundle. The fluorescence emitted by the sample is collected at the end of the fibre and propagated to a camera.

Among the different optical-fibre probes used in fluorescence (Figure 9a), there are approaches based on classical and simple single-pixel measurement (one-excitation and one-collection fibres) [120] and multipixel measurement [134,135], including fibre-based optical endomicroscopy (OEM) [132,136] with potential for depth mapping [132].

Within the latter field, there are high-resolution imaging fibres. An imaging fibre comprises thousands of cores (Figure 9b), each of which acts as a pixel, transmitting the corresponding part of the fluorescence image located at the distal end of the fibre [132,137]. A typical fluorescence image setup is depicted in Figure 9c. High-resolution imaging with these fibres is achieved by minimizing the distance between the cores (centre-to-centre pixel spacing ranges approximately from 3 to 10 µm). However, this distance is limited by a cross-coupling signal between the cores. There are different ways to reduce cross-coupling and thus avoid resolution degradation: high refractive-index contrast between cladding and cores, air-separated cores, or fibres with absorbant cladding material, among others. However, these are all options with high manufacturing costs, limiting their practical use.

Stone et al. developed (in 2017) low-contrast index imaging fibres with adjacent cores of different sizes [137]. For the hexagonal and square array fibre, these fibres have up to 12,247 and 8100 cores, respectively. They used mass-produced materials, such as multimode telecommunications preforms (OM1 PCVD), which makes their potential use in practice low-cost. Building on this study, in 2020, Parker et al. analysed the effect of cross-coupling on the 8100-core square-array imaging fibre [138]. In addition, a novel multifunctional endoscopic fibre based on this CFB was developed in 2018 [136,139,140]. The low-contrast imaging fibre (450 μm corner-to-corner field of view) also contains two capillary channels for fluid delivery/extraction. This fibre was used to detect fluorescently labelled bacteria in lung tissue in ex vivo human lungs.

In 2017, Lukic et al. developed an endoscopic multimode fibre probe for nonlinear imaging, such as anti-Stokes Raman scattering (CARS), second harmonic generation (SHG), and two-photon excited autofluorescence (TPEF) [141]. The design, compact and free of moving parts, is intended for in vivo tissue investigations. A GRIN lens collimates the excitation laser light from the imaging fibre, passed through a long-pass filter and a diffractive optical element, and is focused by a final GRIN lens. On the other hand, the sample signal is collimated by the last GRIN lens, deflected by a prism mirror, and focused by another GRIN lens on the collecting fibre. In any case, there are recent examples of using fluorescence-based optical-fibre probes for cancer detection [142], for instance, by pH sensing [143]. Also noteworthy is a study from 2020, that detects β-sheet amyloid fibrils associated with neurodegenerative diseases [144].

### 4.2. Modified Fibre-Optical Probes

In recent years, numerous articles have addressed the difficulties in controlling some parameters in catheters and needles in the medical field (e.g., shape, force). Fibre Bragg gratings and Fabry–Pérot systems are the most used techniques. The following section summarises a selection of the latest advances (last five years) on modified optical probes based on these two techniques. A brief state-of-the-art is included at the beginning of each section.

#### 4.2.1. Fibre Bragg Gratings

Fibre Bragg grating is the most used technique for shape and temperature sensing, enabling controlled navigation in endoscopy and measurement of the catheters’ position. In addition, this technique is also used for shape and force sensing of medical needles that have a simple bending profile. The combination of various FBGs allows for the calculation of the curvature and direction angle. Some pioneering works surged at the end of the 2000s. Miniaturised fibre-optical sensors based on FBGs were proposed to be integrated into robotics for force feedback [145], and endoscopes for shape detection [146]. After that, the contributions have grown considerably, one of the most typical configurations is the combination of three fibres arranged over the structure [147,148,149]. The error that the core geometry can introduce can be surpassed with multicore fibres [150]. Besides, other geometries have been investigated [151]. For shape reconstruction, there are three main approaches proposed by Roesthuis [149], Moore [150], and more recently by Cui [152].

Nowadays, several examples of FBG sensors for shaping have been investigated for their use in different medical areas. For example, an optical-fibre FBG sensor is proposed for possible use in retinal microsurgery [153]. This type of surgery requires precise control of the instruments in the eye’s interior (due to the delicate tissue in this area). The advantage of this proposal is the axial force-sensing function thanks to the three degrees of freedom (DOF) force sensing microneedle sensor. The sensor uses three optical fibres with dual FBGs and a custom algorithm to decouple the axial and transverse forces. The fibres are attached longitudinally onto the surface of a nitinol tube. This configuration allows the introduction of an injection catheter by the remaining hole and measurements with resolutions of 0.124 mN for transverse forces and 0.74 mN for axial forces (stable results between temperatures of 35 to 37 °C).

Another similar work can be found in [154], in which three fibres with FBGs measure the three-axial force. The main difference is that two fibres are located eccentrically around the structure, and the other one is placed at the resulting hole at the centre of the structure. The device is designed to work in endoscopic surgical robots giving haptic feedback to the surgeon (it could also be used in laparoscopic robots or catheters). A lateral force sensitivity of 838.386 pm/N is demonstrated with a measurement resolution of 1.19 mN. The maximum error was only 6% in a commercial force sensor (Nano17).

Also based on FBGs, the sensor proposed in [155] is designed to detect the interaction forces between the catheter tip and the tissue. The authors propose a 3D printed sensor consisting of a hollow core cylinder, which detects lateral forces. In this case, four optical fibres are sued to decouple the force and temperature parameters. A minimum resolution of 0.52 mN for lateral force (between −0.8 to 0.8 N) and 0.63 mN for axial force (between 0 −0.8 N) is demonstrated. An error below 6.5% is obtained for temperatures ranging from 25–50 °C. A similar device (4 optical fibres) was demonstrated with a maximum shape error of 3.43% [156] and with 1.05 mm error [157].

Another recent proposal for the detection of catheter tip–tissue interaction force can be found in [158]. In this case, the authors combine a symmetrical force-sensitive flexure-based catheter distal sensor with four FBGs for force and temperature decoupling and detection. Thanks to this, the proposed design has improved resolution and sensitivity than reflection-based sensors, avoiding the FBG limitations (chirping failure and low repeatability). In addition, in vivo experiments (cardiac catheterisation) demonstrate a resolution of around 4.6 mN within the measurement range of 0 ∼ 3.5 N [158,159]. Finally, FBGs have also been proposed to measure the deflection angle for vascular access [160]. The device is based on three FBG sensors on an 18 G vascular access needle (90 mm long). The preliminary test has shown a 40° range of where the artery may be located.

**Figure 10:**
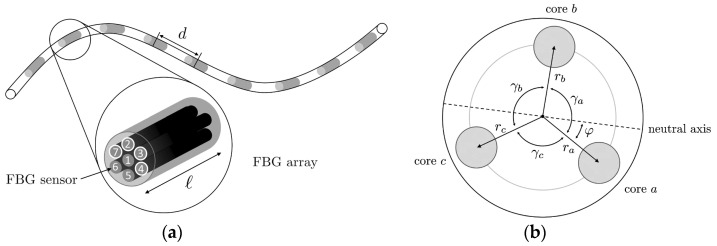
(**a**) FBG system with centre-to-centre distance *d* and sensor length *l*. Numbers represent the different cores: here, the cores of configuration (2347) are highlighted. (**b**) The cross-section of a triplet configuration. Reprinted from [161] under the terms of the Creative Commons Attribution 4.0 International License.

The study of shape sensors using multiple cores can also be found [161]. As shown in Figure 10a, a multicore fibre (seven cores) is used to measure the shape of flexible instruments for endovascular navigation. Different configurations are studied, concluding that at least three linear independent cores are needed for proper 3D reconstruction. It must be considered that the performance of distinct cores varies significantly due to differences in the manufacturing process. An asymmetric configuration, as shown in Figure 10b, can reduce this effect. However, the higher the number of cores used, the better similarity in performance of the cores [161]. The results show an average error of 0.35–1.15 mm and a maximal error of 0.75–7.53 mm over the whole 38 cm sensing length. When the system is used in an endovascular scenario, a maximum error of 2.11 mm is obtained.

A different technique to measure the shape of catheters and needles in the medical field is optoacoustic FBG [162]. An acoustic-optic FBG sensor demonstrates the tracking of the catheter position during interventional magnetic resonance imaging (MRI) [162]. The traditional means to visualise the catheter are the inclusion of magnetic and contrast agents [163], or the inclusion of radio frequency (RF) receiver antennas [164]. The first option can cause obstruction and distortion in the MRI images, whereas the second option can suffer from induced heating in the transmission lines of the RF signals due to resonance effects. For this reason, with this solution, the FBG is used as a dielectric transmission line for the RF signal. The RF signal is converted to an electrical signal by a small coil. The electrical signal is converted to acoustic waves by a piezoelectric transducer, and these waves modulate the reflected light of the FBG. As a result, the measured Bragg wavelength has a linear response concerning RF signal amplitude (flip angle).

**Figure 11:**
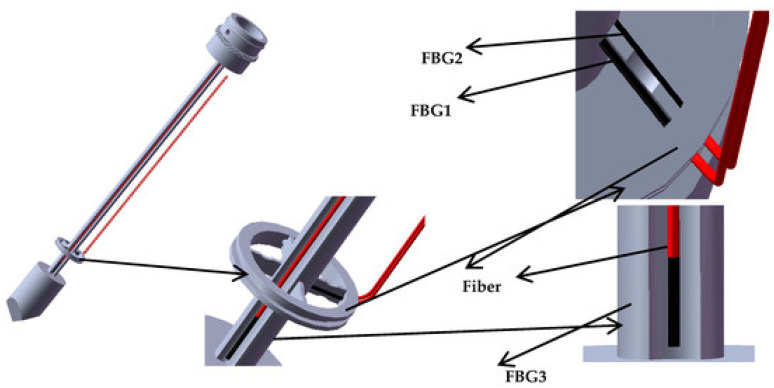
Three groups of FBGs paste position. FBG1 and FBG2 are pasted on the upper and lower surfaces of the new type of elastic beam, while FBG3 is pasted in the groove on the surface of the puncture needle cylinder. Reprinted from [174] under the terms of the Creative Commons Attribution 4.0 International License.

In addition, FBG can also be used to measure puncturing force and deflection angles. In the past, several designs based on optical fibres (FBGs or reflection methods) were proposed to work in needles and catheters. The proposed applications can be microsurgery [165,166] (FBG); minimally invasive surgery [167,168,169] (reflection); micromanipulation [170] (FBG); or other surgical disciplines [171,172] (reflection) [173] (FBG). The referenced prototypes have shown promising results; despite this, some issues are still to be surpassed. Most force sensors are designed to be mounted in the handle of a minimally invasive surgery instrument. They cannot distinguish the contact force applied at the instrument tip and the contact force at the tissue. Furthermore, they cannot sense the torque change but only sense axial force during puncture surgery. Focusing on FBG sensors, in the last five years, some proposals have improved previous results. For example, in [174], a new type of FBG sensor for measuring the puncturing force in needles is proposed and demonstrated. The sensor uses a reference fibre method implementing three FBGs. As shown in Figure 11, the first fibre and the second reference fibre are placed on the upper and lower surfaces, and the third fibre is placed over the puncturing needle cylinder. With this configuration, the torque is estimated by the difference of Bragg wavelength between first and second fibres. The axial force is measured by the difference between the Bragg wavelength of the third and second fibres. This sensor has the advantage of distinguishing axial forces at the tip and tissue (sensitivity of 0.089 nm/N) as well as changes of torque (average sensitivity of 22.8 pm/mN·m).

Carotenuto et al. proposed FBG sensors in epidural procedures [175]. The system was tested with real tissue [176], providing a preliminary indication of the potential of this technique to localise the epidural space. Some related works can be found in [177,178].

Finally, a multiparameter measurement FBG-based needle is proposed in [179]. In this case, the FBG fibre is made sensitive to chemical parameters by a polymer functionalisation of the fibre optic. Specifically, the authors make the FBG sensitive to relative humidity, resulting in a Bragg wavelength shift of ~0.38 nm for relative humidity changes from ~20% to ~90%. In addition, the FBG-based needle sensor can measure the breathing rate during mechanical ventilation, allowing the relative humidity change during this process to be followed.

#### 4.2.2. Fabry–Pérot Interferometer Sensor

The sensitivity to lateral forces is considerably higher than axial forces due to the axial rigidity of the fibre. FBG sensors have been proposed to measure both forces, as described previously. However, a better solution to measuring axial forces is accomplished by Fabry–Pérot-based sensing. Fabry–Pérot interferometer (FPI) sensors have been used for several applications, e.g., needle insertion force [180,181], microsurgery [182] or minimally invasive surgeries [183].

A demonstration of the integration of an FPI sensor into an 18-G needle has been reported [184]. The sensor has good accuracy (maximum error of 65 mN/10 N) and possesses an intrinsic low cross-sensitivity to temperature (12 mN/°C). However, the strength required for placing the sensor in smaller diameter needles may be not sufficient. For this reason, the authors recommend placing the FPI sensor on a microcapillary tube (e.g., Invar capillary) or a thin polyimide film.

**Figure 12:**
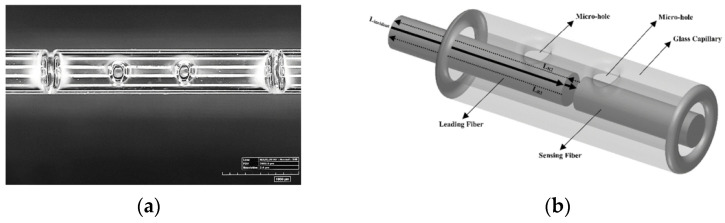
(**a**) The two microholes were created on a borosilicate glass capillary housing by the laser cutting process. (**b**) The final design of the custom FPI based force sensor. Reprinted with permission from [186] © The Optical Society.

Later, an FPI sensor is proposed for prostate biopsy needle interventions under MRI [185]. In this case, the cavity is different from previous proposals made by a hole in a glass capillarity. Two single-mode fibres encapsulated in the glass and at both sides of the hole are used as semi-reflecting mirror surfaces (authors propose coating to improve the signal-to-noise ratio and resolution). They measure an axial force up to 20 N (higher than typical values in prostate interventions). In 2019, the same authors published an extended article with a complete set of the custom FPI sensor integrated into an 18-G nitinol biopsy needle. The sensor slightly differed from the previous proposal mentioned above, as the fibres are fixed in the capillary by two microholes (Figure 12a). In contrast, the cavity is formed by the distance between fibres (Figure 12b). The FPI sensor has been tested in a prostate phantom under real-time MRI guidance [186]. The FPI sensor has a force measurement range of 0–13 N with 0.1 N resolution. An improved version was published in 2020. The surfaces of the single-mode fibres were coated with Ti, obtaining a measurement range of up to 20 N and a resolution of 0.03 N [187].

Using a similar concept but in a multimode fibre, a FPI-based sensor is demonstrated to measure the needle curvature and temperature [188]. A maximum sensitivity of −0.152 dB/m^−1^ with a resolution of 0.089 m^−1^ is obtained for curvature. The resulting cross-sensitivity to temperature is small.

Finally, FPI sensors for MRI-guided robotic systems have also been presented in [189] (which is an optimised version of [180]). One challenge of this sensor is the loss avoidance of the tactile sensation of tool manipulation. One solution is to use force sensors to measure the insertion force and use haptic devices to display to the clinician. One of the main challenges for using sensors in MRI systems is the emission of RF signals. For this reason, this application is suitable for FPI sensors. Furthermore, due to the small size of FPI sensors, they could be integrated into tiny biopsy needles.

## 5. Summary and Conclusions

A review of the latest results on biomedical photonic sensors based on optical fibres has been presented. The articles were classified into different categories. First, the BOFSs focused on measuring physical (vital signs, tissue temperature) and biochemical parameters (oxygen, pH, cancer-related and others) were presented. Second, the first publications towards the evaluation of lab-on-fibre technology for medical applications were described. Third, some novelties in the use of photonic or optical probes in medicine were covered.

There has been significant novelty in optical-fibre sensors since the last reviews on biomedical applications of optical sensors were published. Examples of outstanding optical-fibre sensor performances, new methods, optical probes, and sensing strategies have been presented and detailed below.

Spatially resolved temperature measurements have reported several technical advancements with a strong focus on real-time temperature monitoring of tissue laser ablation. For instance, the continuous temperature control during laser ablation, including preplanning (modelling) information, has been reported in a real application (interstitially) with excellent results. Other works have tackled the measurement of the dynamic response of temperature of the sensors and 3D temperature mapping. Most sensors use FBGs, and a comparison between different modulation techniques of transducers has also been reported. Regarding vital-sign monitoring, a comparison of other sensing techniques, and potentially low-cost optical sensors tested in a clinic environment (based on specklegram) have been reported to monitor heart rate and motion of patients. Furthermore, fibre-optic respiratory sensors incorporated into oxygen delivery devices and sense humidity have been developed and demonstrated. The higher level of maturity in physical parameter monitoring is exemplified as most articles include systems combining different sensors and medical instruments in real-time applications. We identify cases in which optical-fibre sensors directly meet a need in biomedical applications, for instance, capillary refill measurements.

Regarding biochemical parameters sensing, in particular pH sensing, optical-fibre sensors insensitive to bending and very high sensitivities have been reported. Outstanding optrodes for pH and oxygen sensing on multicore functionalized fibres were reported, in which each core could act as an individual transducer. Recently, time-resolved measurements were proposed as an additional tool providing insight into the role of the density of fluorophores, and in general, on fluorescence dynamics. Regarding oxygen, a novel interstitial diffuse optical probe combined with diffusion transport-based modelling allowed the biological composition of tissues to be retrieved, thus enabling the characterization of blood fraction and oxygen saturation in tissue. This method has strong potential for use during light-related therapies. Furthermore, alternative approaches based on SPR and oxygen transport mechanisms through haemoglobin have been reported for dissolved oxygen sensing. Other biochemical parameter sensors were covered, primarily based on the plasmonic resonance effect, including biorecognition elements and innovative strategies with a few tested in vivo. Some offer excellent sensor properties, outperforming, in some cases, traditional sensors.

Optical probes for endoscopy based on Raman backscattering measurements have been reported, incorporating separate fibres to avoid undesired Raman signals and pH detection in an ex vivo ovine lung model. Fibres for spectroscopy imaging have been developed, including outstanding breakthrough developments that allowed depth mapping and nonlinear imaging. Optical probes for shape sensing (primarily based on FBG and FPI) have been tested inside needles (including multiparameter sensing) and catheters. Applications ranging from sensing between tip and tissue to MR-guided robotic systems, among others, were used on prostate phantoms in real-time under MR guidance.

Overall, the enormous diversity of optical-fibre sensors has been enlarged with the introduction of new capabilities for sensing. For example, besides using metal coatings or NPs to exploit SPR and SERS effects, they have been combined with other structures (e.g., FBG or tilted FBG) to provide further flexibility and opportunities in the biomedical field. On the other hand, biosensors have strong potential for the diagnosis/identification of biomarkers for cancer, and optical-fibre flow cytometers have been remarkably attempted. Thus, biosensors show a pathway towards achieving a better detection limit for many biochemical parameters and targets. However, just a few examples can be found for in vivo applications. Hence, a higher number of studies and an increase in the statistics are needed to test these sensors.

In general, multiparameter-sensing platforms are pursued, including lab-on-fibre devices. In this regard, several multiparameter optical-fibre sensors have been demonstrated with potential for biomedical applications. Moreover, a couple of recent examples towards lab-on-fibre devices have been reported. It is a promising approach since it could allow a better understanding of biophysical processes by simultaneously measuring several parameters in real time. Upon these demonstrations, it can be expected that multiparameter sensing in a single fibre, and the implementation of multiple sensors and multiplexing techniques into a single device, could potentially reduce the cost of the systems.

## Figures and Tables

**Table 1:** Summary of characteristics of most optical-fibre sensors described in Section 3, including some remarks and highlights of other reference sensors found in the literature. LOD: limit of detection; t_r_: rise time; t_f_: falltime; PBM: p-mercaptophenylboronic acid.

Measurand	Sensing Strategy	Sensor Performance	Remarks/Highlights
Dissolved oxygen [82]	SPR-based Gold coated	Sensitivity: 8.89 nm/(mg/L) Range: 2–20 mg/L LOD: 0.01 mg/L	New method based on refractive index changes induced by oxyhaemoglobin modifications. Improved sensitivity with carbon nanotubes
Blood volume and oxygen saturation [85]	Diffuse reflectance +modelling	-	Interrogation for deep-seated tissue to measure dynamic tumour hypoxia In contrast to commercial products (Oxylite, Eppendorf), it averages over the microvascular spatial scale of 3 mm (oxygen content may vary orders of magnitude in micrometre scale) Enables long duration and high time-resolution measurements For hypoxia studies in cancer research
pH	Various (not reviewed in this work)	Optical-fibre-based (See [73] and references therein): Lowest t_r_/t_f_: 12 s/22 s Resolution down to 0.0022 (pH 3–7) Max. range 1–12 Commercial [74]: Response (t_90_) < 60 s Ranges: 5–7 Resolution: 0.003 at 6 pH Drift < 0.005/day	-
pH [78]	Ratiometric fluorescence Multicore plastic fibre Hybrid sol–gel sensitive material	Max. Resolution: 0.0013 pH (6–8 pH unit range) Drift: 0.003 pH/22 h (0.004/h in vivo) Response t_90_ < 2 min	Tested in in vivo human tissue Characterization (long-term stability, temperature and ionic strength cross-sensitivity, biocompatibility) Ratiometric approach (resistant to laser power fluctuations) Outperforming state-of-the-art pH sensors
pH and oxygen [79]	Multicore Fibre Fluorescein-based sensors Ratiometric fluorescence	Accuracy: ±0.02 pH 0.6 mg/L (oxygen)	Ex vivo ovine lung tumour model For deep-in-tissue measurement 19 cores that could act as independent measurement channels
pH [76]	Ratiometric fluorescence Tapered fibre Dye in hybrid sol–gel matrix	Accuracy: ±0.04 pH Drift: 3.2% after 92 min Range tested: 4–8 pH	Ex vivo aqueous humor samples Cataract surgery 56 samples from 50 patients
pH [73]	U-shape fibre Spectroscopic Ethyl cellulose film with sol–gel matrix	Accuracy: ±0.2 pH Range: 4.5–12.5 pH Resolution: 0.02 pH t_r_/t_f_: 45 s /46 s Low temp.sensitivity (21–39 °C)	Tested in human serum Dye free
Cancer biomarker (cytokeratin 17) [89]	SPR+tilted FBG Gold coated Encapsulated in a porous gel matrix to mimic tissue samples	Sensitivity: 1.5 dB at 10^−10^ g/mL LOD: 1 pM (liquid samples)	Ex vivo human lung tissue Confirmed selectivity and specificity to target proteins Stable SPR signal in a nonliquid sample Pathway for in situ and on-line detection of biomarkers in tissues
Breast cancer cells [90]	AptasensorSPR+tilted FBG Gold coated Gold NPs MAMA2 aptamers	Sensitivity: 100 cells/mL 10 cells/mL (using gold nanoparticles) LOD: 49 cells/mL	Detection of rapid circulating breast cancer cells in vitro at very low concentrations Label free Direct and real-time detection of targets Sensitivity comparable to other methods (e.g., THz chemical microscopy)
Breast cancer HER2 biomarker [92]	SPR Gold coated HER2 aptamers/proteins/antibodies on fibre surface	LOD: 1 µg/mL (around 8 nM) Amplified to 10 ng/mL (around 86 pM) using HER2 antibodies	Tested in phosphate buffer saline Label-free sandwich assay Includes a study of influence of gold film thickness on the refractive index sensitivity
Cells and particles [93]	Double-clad fibre Fluorescence Scattering	Flow rate up to 800 µL/min 2500 particles/s(commercial 10,000 of particles/s or more)	Tested in HCT 116 colon cancer cells All-silica fibre-based microflow cytometer High performance towards miniaturization Robust, portable, low cost
PfHRP2 [63]	SPR Gold coated Covalent bonding of antibodies to fibre surface	LOD: 1 µg/mL (improvable by a factor of 10) Usual rapid diagnostic tests reported 0.8 ng/mL	Tested in vitro Towards avoiding paper-based dip sticks leading to false-negative results Not the highest sensitivity Multiplexing system for detection of different proteins (e.g., LDH and HRP2)
Glucose [65]	Plasmonic tilted FBG Self-assembled PMBA and monolayer and gold NPs	Range: 1 nM–10 mM Max. Sensitivity: 1.08 dB/nM LOD: 295 pM Low temperature cross-sensitivity	Validated in simulated human saliva High sensitivity and preferential selectivity of glucose over fructose and galactose Outperforms most glucose sensors [65,66] Novel mechanism for glucose detection
Calmodulin [64]	Plasmonic tilted FBG Ge-doped fibre Gold coated Gold mirror on tip	LOD: 0.44 nM	Reflection probe Microliter volume detection small-size sensor
Cyt c [67]	Multimode optical fibres SPR Gold NPs on the fibre facet	Max. Concentration: 80 µM LOD: 60 nM (comparable to electrochemical methods)	Validated in bovine serum Portable and low-cost platform Focused on absorption changes
*E. coli* O157:H7 [68]	Aptasensor Microcavity MZ Peptide aptamers APTES/glutaraldehyde	10 colony-forming units per mL	Label-free real-time measurements Similar LOD to other optical-fibre platforms and other detection methods Verified against ELISA method One-step femtosecond laser fabrication

## Supplementary Materials

The following are available online at https://www.mdpi.com/article/10.3390/s21196469/s1, Figure S1 includes the articles published per year about optical fibre sensors found in the Web of Science Core Collection database. Figure S2 corresponds to a network map of the most common keywords in publications linked to optical fibre sensors. Table S1: Extended list of latest reviews on optical-fibre sensors. Most reviews are from 2018, including several directly linked to biomedical applications or with biomedical interest.
