# Supplementary material for: Recent Advances in Biomedical Photonic Sensors: A Focus on Optical-Fibre-Based Sensing
Source: Sensors (Basel). 2021 Sep 28;21(19):6469. doi: 10.3390/s21196469 (PMC8513032; doi:10.3390/s21196469)
Supplement: Supplementary file 1 [file sensors-21-06469-s001.zip › sensors-1387900-supplementary.pdf]

Supporting Information

Recent Advances in Biomedical Photonic Sensors: A Focus on Optical Fibre-Based Sensing

Mario Ochoa [1,2,3,*], José Francisco Algorri [1,2,3], Pablo Roldán-Varona [1,2,3], Luis Rodríguez-Cobo[3] and José Miguel López-Higuera[1,2,3*]

[1] Photonics Engineering Group, University of Cantabria, 39005 Santander, Spain; mario.ochoa@unican.es (M.O.); pablo.roldan@unican.es (P.R.-V.); lopezhjm@unican.es (J.M.L.-H.)
[2] Instituto de Investigación Sanitaria Valdecilla (IDIVAL), 39011 Santander, Spain
[3] CIBER-bbn, Institute of Health Carlos III, 28029 Madrid, Spain; luis.rodriguez@unican.es
* Correspondence: mario.ochoa@unican.es

1. Bibliometric Data

Web of Science (WoS) Core Collection database was used to retrieve published articles related to optical fibre sensors. Since some authors write fiber or fibre, optical or optic, fibre-optic or fiber-optic, sensor or sensing, etc., the combination set of keywords linked to optical fibre sensors is large. Hence, we attempted to include the most important combinations with the following query:

"((TI=(Optical fibre sensor)) OR TI=(Optical fiber sensor)) OR TI=(optic fibre sensor)) OR TI=(optic fiber sensor)) OR TI=(fiber optic sensor)) OR TI=(fibre optic sensor)) OR TI=(optical fibre sensing)) OR TI=(optical fiber sensing)) OR TI=(optic fibre sensing)) OR TI=(optic fiber sensing)) OR TI=(optical fiber probe)) OR TI=(optical fibre probe)) OR TI=(optic fibre probe)) OR TI=(optic fiber probe)) OR TI=(fibre optic probe)) OR TI=(fiber optic probe)) OR TI=(optical fibre biosensor)) OR TI=(optical fiber biosensor)) OR TI=(optic fibre biosensor)) OR TI=(optic fiber biosensor)) OR TI=(fibre sensor)) OR TI=(fiber sensor)) OR TI=(fiber-optic sensor)) OR TI=(fibre-optic sensor)) OR TI=(fibre bragg grating)) OR TI=(fiber bragg grating)) OR TI=(fbg)) OR TI=(fiber bragg grating (fbg))) OR TI=(fiber Sagnac interferometer)) OR TI=(fibre Sagnac interferometer)) OR TI=(fibre Mach-Zehnder)) OR TI=(fiber Mach-Zehnder)) OR TI=(fibre Fabry-Perot)) OR TI=(fiber Fabry-Perot)) OR TI=(optic fibre interferometer)) OR TI=(optical fibre interferometry)) OR TI=(optical fiber interferometry)) OR TI=(optic fiber interferometer)) OR TI=(optical fibre interferometer)) OR TI=(optical fiber interferometer)) OR TI=(optical fibre distributed)) OR TI=(optical fiber distributed)) OR TI=(optic fibre distributed)) OR TI=(optic fiber distributed)) OR TI=(fiber optic distributed)) OR TI=(fibre optic distributed)) OR TI=(fibre grating)) OR TI=(fiber grating)) OR TI=(long period fiber grating)) OR TI=(long period fibre grating)) OR TI=(fibre long period grating)) OR TI=(fiber long period grating)) OR TI=(optical fibre aptasensor)) OR TI=(optical fiber aptasensor)) OR TI=(fiber optic aptasensor)) OR TI=(fibre optic aptasensor)) OR TI=(optic fibre aptasensor)) or TI=(optic fiber aptasensor)) OR TI=(optic fiber aptasensor)) OR TI=(optic fibre aptasensor)"

We note other modifications or inclusions into previous query did not retrieve significant variations. Such query was accessed on 17.09.2021 retrieving 23895 records. In addition, a refinement was performed in the WoS engine, including several types of sensors or keywords related to specific sensors, as depicted Figure S1 below.

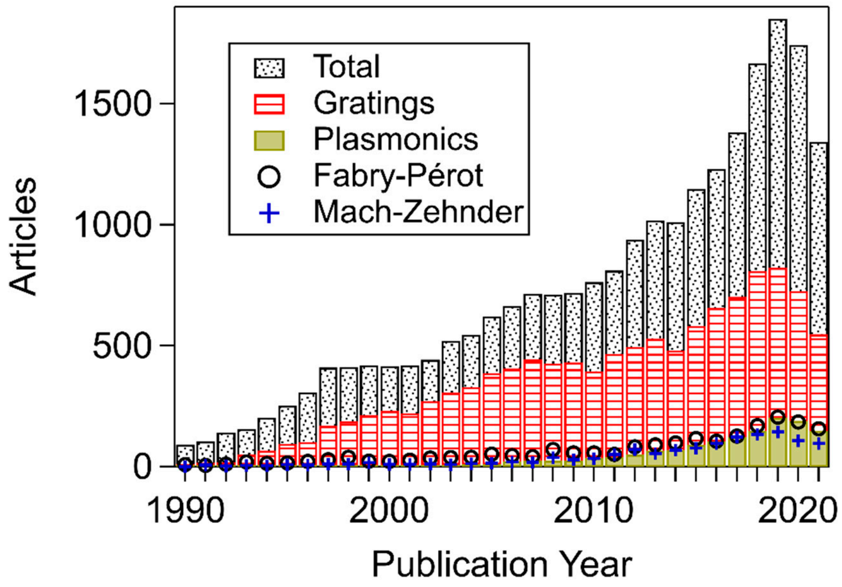

Figure S1. Articles as a function of publication year, including some representative types of optical fibre sensors.

Then, to generate the network map of Figure S2 (below), all 23895 records were loaded into VOSviewer software, and some data cleaning was performed. Some authors could write keywords differently such as fibre, fibres, fiber, etc. All these have the same meaning. Thus, such keywords were replaced and merged into a single keyword. For example, we merge "fibre bragg sensor", "fiber Bragg sensors", or "bragg grating sensor", etc., into a single term "fbg" as shown below. This procedure was applied to several keywords, not only fibre. Finally, the map network includes keywords with more than two hundred appearances.

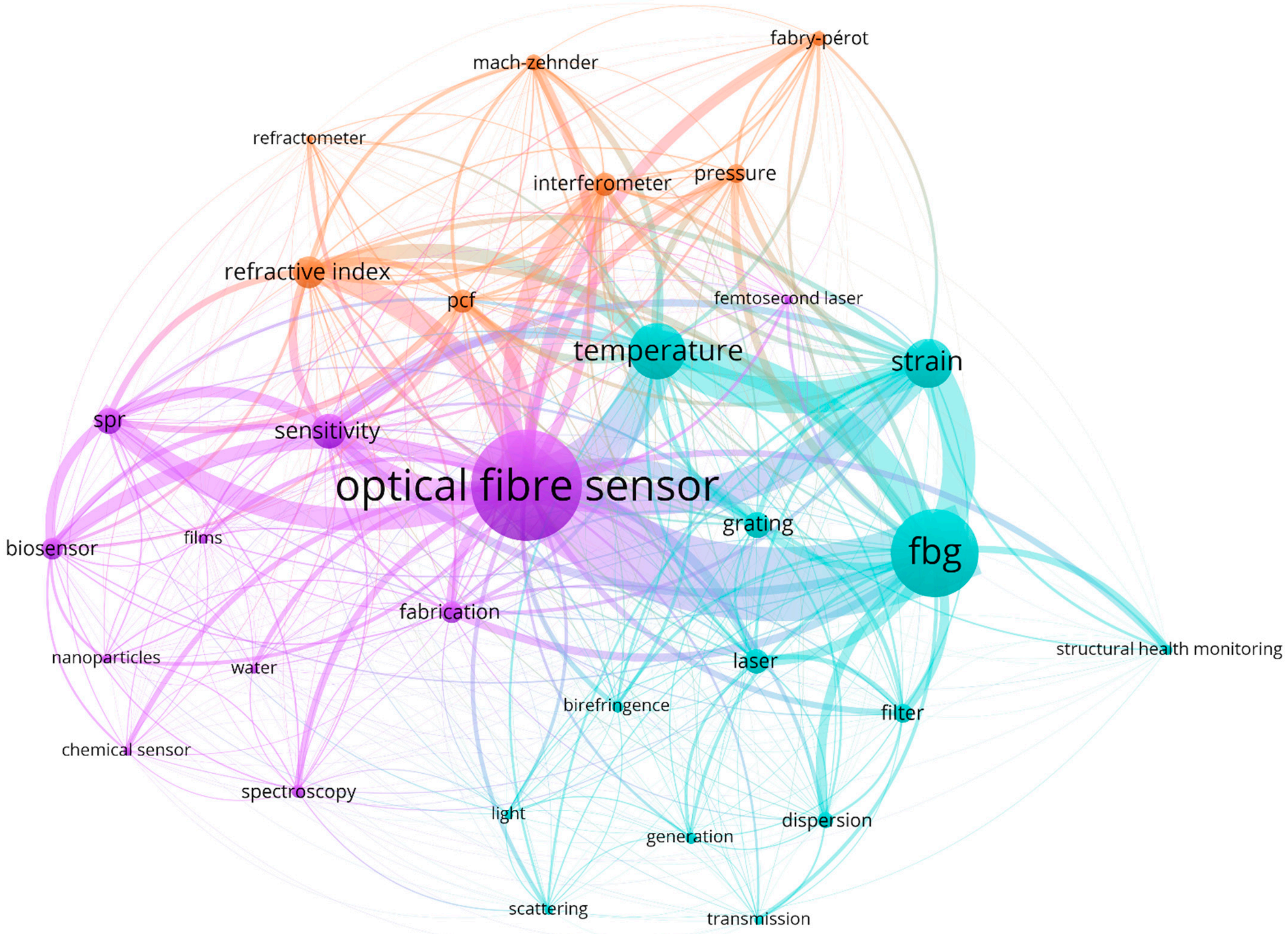

Figure S2. Map visualization of keywords links strength network built from 23 895 articles found in Web of Science Core Collection when searching for optical fibre sensors (accessed on 17/09/2021). Only keywords exceeding two hundred occurrences are depicted. The period selected is from 1965 to 2021. Abbreviations. Surface plasmon resonance: spr; fibre Bragg grating: fbg; photonic crystal fibre: pcf. Graph created using VOSviewer software version 1.6.17 [1].

A similar procedure was applied to the generation of Figure 1 within the manuscript. In this case, the results from the previous query were refined by searching "biomedical", "medical", "medicine", "biomedicine" words in the WoS engine. Such refinement retrieved over 2000 records.

However, this list includes duplicated records that were deduplicated using Zotero. As a result, 1351 records were retrieved and used to generate Figure 1. For Figure 1b, only keywords with more than 20 occurrences are shown.

2. Compilation of latest published reviews related to optical fibre sensors

Table S1. List of latest reviews (not complete list and most from 2018) and roadmaps on photonic/optical fibre sensors (mostly optical fibre). The majority of reviews listed consider the fibre acts as the transducer in a photonic sensor. The first three categories mainly focus on biomedical applications, whereas the rest have a biomedical interest for optical fibre sensors.

Category	Topic	Content	Year	Ref.
General for Biomedical applications	Optical devices for biomedical sensing	Principles of operation, advantages, disadvantages of several techniques and platforms (optical biosensors, vertical grating couplers, plasmonic sensors, surface plasmon resonance optical fibre biosensors, and metasurface biosensors, Photonic crystal-based biosensors, thin metal films biosensors, and fibre Bragg grating biosensors).	2021	[2]
	Biomedical optical fibres	Emerging medical and health-field applications of optical fibres, including the fabrication of implantable devices, wearable sensors, and photodetection and therapy setups. A small section for optical fibre sensing, including pH, strain, temperature on doped, FBG fibres.	2020	[3]
	Fibre-Optic Technology for Point-of-Care Diagnosis and In Vivo Biosensing	Advances in biosensors incorporated into optical fibres.	2020	[4]
	Optical sensors for biomedical diagnosis	Recent progress of optical sensors for biomedical diagnosis including: surface plasmon resonance, localised surface plasmon resonance, evanescent wave fluorescence, bioluminescence among others.	2020	[5]
	Biosensors and techniques for enhanced sensing ability	Overview of differentiating factors of fibre-optic biosensors, which are tailored for specific health needs. Alterations made in biosensing elements, including pH elements, enzymatic elements, as well as those sensors utilizing antibodies and whole-cell bacteria	2019	[6]
	Healthcare using polymer optical fibres (POF)	State of the art developments for sensors with applications on movement analysis, physiological parameters monitoring, instrumented insoles, instrumentation of healthcare robotic devices such as exoskeletons, smart walkers, actuators, prostheses, and orthosis.	2019	[7]
	Biomedical application of optical fibre sensors	Overview type of sensing for medicine. Covers specific parameters in health: Physical, chemical and biological	2018	[8]
	Optics in medicine	Historical aspects of the role of optics in medicine summarizing current technologies and future trends. Sections on fibre bundles, fibrescopes and endoscopes, among others, are included.	2016	[9]
	Optical fibres in biomedical research and clinical practice	Background to understand how optical fibres function (including different categories of fibres), and show their use for biomedical photonics applications. A small section of optical fibres sensing for biomedical applications is provided.	2014	[10]
By parameters	Vital signs monitoring	Brief theory of optical fibre sensors and summary of research works on vital signs monitoring. Methods and techniques to improve sensitivity that provides stability and resistance (doping or coating) to protect from external factors for in vivo applications are included. Most operate through FBG and LPFG and interferometers	2021	[11]

	Oxygen & pH	Discussion of representative techniques (including fibre optic sensors) that have reached commercial development is provided.	2021	[12]
	Shape sensors	Review the current state of the art of fibre optic shape sensors based on optical multicore fibres with embedded strain sensors. A section devoted to medical applications is provided	2021	[13]
	Glucose	Comparison of different types of optical fibre sensors for glucose in biomedicine.	2021	[14]
	DNA	Comparison of technologies including sensitivity and detection limits for DNA-based sensors	2021	[15]
	Biomolecules detection	Optical techniques-based sensors (including optical fibres) for the detection of different biomolecules (such as proteins, nucleic acid, glucose, DNA, cholesterol, uric acid, amino acids, and dopamine) and microorganisms have been discussed	2021	[16]
	Cardiorespiratory	Recent advances on cardiorespiratory monitoring using FBG sensors. Overview of the working principle, performance and configuration.	2020	[17]
	Analyte sensing using photonic crystal fibre (PCF)	A brief overview of the development of analyte sensors based on photonic crystal fibre (PCF). Recent advances in particular areas of gas sensing, chemical species and bio analytes are presented.	2020	[18]
	Temperature	Reviews achievements in temperature optical fibre sensors, different configurations of the sensors reported over the last five years, and application of this technology in biomedical applications	2020	[19]
	Force	Overview of MRI-compatible fibre-optic force sensors based on different sensing principles, including light intensity modulation, wavelength modulation, and phase modulation for MRI-Guided Interventions and Rehabilitation	2017	[20]
	Pressure	Reviewing the current state-of-the-art of optical fibre pressure sensors with suitability for medical applications with particular reference to in vivo measurements, e.g. for urodynamic and cardiovascular assessment	2015	[21]
	Physiological	Review of applicable technologies and relevance to use during magnetic resonance imaging procedures	2015	[22]
By sensing technique	Biosensors in cancer detection – Surface Plasmon Resonance (SPR)	Review of surface plasmon resonance biosensors between 2005-2020. Focus on testing the effectiveness of cancer markers and the discovery of new cancer markers.<br>Works on the determination of micro RNA and large particles such as vesicles, exosomes and cancer cells are covered.	2021	[23]
	Long-period fibre Bragg grating (LPFBG): Label-free biosensors	Review of the state-of-the-art LPG-based label-free biosensors, including future research trends	2021	[24]
	Biosensor technologies in medicine	Detection of biochemical markers and research of molecular targets	2020	[25]
	Fibre Bragg grating (FBG) for biomedical applications	Review of the FBG-based measuring systems, their principle of work, and their applications in medicine and healthcare.<br>Particular focus on biomechanics, minimally invasive surgery, physiological monitoring and medical biosensing	2020	[26]
	SPR (cancer detection)	Review of 71 papers (2005-2020, mostly in the last decade) on surface plasmon resonance biosensors for cancer detection with emphasis on effectiveness of caner markers and discovery of new cancer markers.	2020	[23]
	SPR	An overview of the advancements in surface plasmon resonance technology considering smart layers design, multiplexing concepts, continuous monitoring and in vivo sensing	2020	[27]
	SPR	Research status of fibre optic biosensor based on SPR	2019	[28]

	SERS-active photonic crystal probe	Review on most promising recent biosensors based on PCFs, including hollow-core PCFs, suspended-core PCFs and side-channel PCFs, with focus on challenges for translation into clinically viable next-generation sensitive biopsy needle sensing probe	2019	[29]
Lab-on-Fibre	Stimuli-responsive materials for smart Lab-on-Fibre optrodes	Recent progress in the realisation of optical devices integrated with stimuli-responsive materials (e.g. hydrogels, microgels, polymer brushes.) sensitive to temperature, pH, electric and magnetic fields or light, with a special focus on optical fibre-based probes.<br>Biomedical interest: Bio-chemical sensors, light-triggered loco-regional drug delivery systems	2021	[30]
	Lab-on-Fibre roadmap for Multifunctional Plug and Play Platforms	Overview of lab-on-fibre technology, including fabrication techniques, prototypes and merging of LOF techniques with optomechanics.<br>Biomedical interest: Description of optical fibre probes for Optical Coherence Tomography catheters, for mapping tissue mechanics and for ultrasound applications	2020	[31]
	Lab-on-fibre: Plasmonic nano-arrays for sensing	Review of nanofabrication technologies for optical sensors mainly integrated on optical fibre tip. Plasmonic nano-arrays and sensors are compared and analysed. Main research directions and progress are summarised.<br>Biomedical interest: Potential for biochemical detection and incorporation into needles.	2020	[32]
	Multifunctional integration on optical fibre tips	A broad overview of fabrication technologies for patterning optical fibre tips and typical structures integrated on them.<br>Biological interest: All-optical Ultrasound transducer for medical imaging in vivo	2020	[33]
	Lab-in-a-fibre sensors: A review	Principles of the Lab-in-a-fibre approach, including current and emerging technologies for optical fibre sensing. The emerging trends and challenges are described. Finally, a side-by-side comparison with lab-on-chip is presented.<br>Biomedical interest: General	2019	[34]
	Lab on Fibre Technology for biological sensing applications	Strategies, the main achievements, perspectives and challenges of Lab on Fibre devices with special focus on biological sensing applications	2016	[35]
Optical fibre Optofluidics	Fibre Optofluidic Lasing and Sensing	Recent Progress in Fibre optofluidic lasers for sensing focused mainly on optical fibre resonators, gain medium, and the emerging sensing applications.	2021	[36]
	Optical Fibre Optofluidic Bio-Chemical Sensors	Current status and recent progress, including sensing principles, structures and applications.	2021	[37]
	Micro-/Nanofibre Optics	Basics of micro-/nanofibre (MNF) optics presenting current status and progress. Besides, hybrid structures are reviewed by merging MNF with functional structures, including chemical indicators, quantum dots, dye molecules, plasmonic nanoparticles, 2-D materials, and optofluidic chips.	2020	[38]
By fabrication technique	Optical Fibre Integrated Functional Micro-/Nanostructure Induced by Two-Photon Polymerization	Research progress in the last ten years and prospects of micro and nanostructures fabricated by two-photon polymerization and classified by micro-optics, optical waveguide devices and optical micro-cavities	2020	[39]

	Optical fibre sensors by direct laser processing: A review	Most important transducing structures fabricated with direct laser writing are covered.	2019	[40]
	Mach–Zehnder interferometers fabricated by femtosecond laser	Overview of MZI optical fibre sensors with micro-cavity fabricated by femtosecond laser writing	2019	[41]
	Self-assembly on optical fibres for "lab-on-fibre" optrodes	Main fabrication techniques and strategies of lab-on-fibre optrodes based on self-assembly processes on optical fibres	2018	[42]
Other	Interferometer (biosensors)	Review of operating principles, sensing structure and application fields of four types of interferometer biosensors based on specialty fibres: MZI, MI, FPI, SI	2021	[43]
	Optical Resonant (Bio-Chemical)	Recent trends on optical resonant sensors which may involve plasmonic or geometrical resonances	2021	[44]
	Lossy Mode Resonances	Recent advances in lossy more resonance (LMR) fibre optic sensors	2021	[45]
	Bio-chemical (POF)	Review on Biochemical label-free sensing applications including chemical and biological receptors combined with polymer optical fibres	2021	[46]
	Optical Fibre Technologies for Nanomanipulation and Biodetection	Current trends and future direction for applications of optical fibres for nanomanipulation and biodetection. Biomedical interest: General and single-molecule sensing.	2021	[47]
	Optical biosensors	An exhaustive and comprehensive review of optical biosensors including surface plasmon resonance (SPR), optical waveguides, optical resonators, photonic crystals, and optical fibre (examples of medical applications reported)	2020	[48]
	No-core optical fibre sensors	Summary of the sensing applications, challenges and research direction in the development of no-core optical fibre sensors.	2020	[49]
	Roadmap for optical sensors	Roadmap for a variety of optical sensors for several applications. Biomedical interest: One section includes the roadmap for biomedical optical sensors	2017	[50]

References

1. van Eck, N.J.; Waltman, L. Software survey: VOSviewer, a computer program for bibliometric mapping. *Sci. 2009 842* **2009**, *84*, 523–538, doi:10.1007/S11192-009-0146-3.

2. Kazanskiy, N.L.; Khonina, S.N.; Butt, M.A.; Kaźmierczak, A.; Piramidowicz, R. State-of-the-art optical devices for biomedical sensing applications—a review. *Electron.* 2021, *10*, 973.

3. Rezapour Sarabi, M.; Jiang, N.; Ozturk, E.; Yetisen, A.K.; Tasoglu, S. Biomedical optical fibers. *Lab Chip* 2021, *21*, 627–640.
4. Tabassum, S.; Kumar, R. Advances in Fiber-Optic Technology for Point-of-Care Diagnosis and In Vivo Biosensing. *Adv. Mater. Technol.* **2020**, *5*, 1900792, doi:10.1002/ADMT.201900792.
5. Pirzada, M.; Altintas, Z. Recent Progress in Optical Sensors for Biomedical Diagnostics. *Micromachines 2020, Vol. 11, Page 356* **2020**, *11*, 356, doi:10.3390/MI11040356.
6. Mowbray, S.E.; Amiri, A.M. A brief overview of medical fiber optic biosensors and techniques in the modification for enhanced sensing ability. *Diagnostics* **2019**, *9*, doi:10.3390/diagnostics9010023.
7. Leal-Junior, A.G.; Diaz, C.A.R.; Avellar, L.M.; Pontes, M.J.; Marques, C.; Frizera, A. Polymer Optical Fiber Sensors in Healthcare Applications: A Comprehensive Review. *Sensors 2019, Vol. 19, Page 3156* **2019**, *19*, 3156, doi:10.3390/S19143156.
8. Correia, R.; James, S.; Lee, S.-W.; Morgan, S.P.; Korposh, S. Biomedical application of optical fibre sensors. *J. Opt.* **2018**, *20*, 073003, doi:10.1088/2040-8986/AAC68D.
9. Méndez, A. Optics in Medicine. *Opt. Our Time* **2016**, 299–333, doi:10.1007/978-3-319-31903-2_13.
10. Keiser, G.; Xiong, F.; Cui, Y.; Shum, P.P. Review of diverse optical fibers used in biomedical research and clinical practice. *J. Biomed. Opt.* **2014**, *19*, 080902, doi:10.1117/1.JBO.19.8.080902.
11. Mayoral, C.P.; Gutiérrez, J.G.; Pérez, J.L.C.; Treviño, M.V.; Velasco, I.B.G.; Cruz, P.A.H.; Rosas, R.T.; Carrillo, L.T.; Ríos, J.A.; Apreza, E.L.; et al. Fiber Optic Sensors for Vital Signs Monitoring. A Review of Its Practicality in the Health Field. *Biosens. 2021, Vol. 11, Page 58* **2021**, *11*, 58, doi:10.3390/BIOS11020058.
12. Werner, J.; Belz, M.; Klein, K.F.; Sun, T.; Grattan, K.T.V. Fiber optic sensor designs and luminescence-based methods for the detection of oxygen and pH measurement. *Measurement* **2021**, *178*, 109323, doi:10.1016/J.MEASUREMENT.2021.109323.
13. Floris, I.; Adam, J.M.; Calderón, P.A.; Sales, S. Fiber Optic Shape Sensors: A comprehensive review. *Opt. Lasers Eng.* **2021**, *139*, 106508, doi:10.1016/J.OPTLASENG.2020.106508.
14. Perez, J.L.C.; Gutiérrez-Gutiérrez, J.; Mayoral, C.P.; Pérez-Campos, E.L.; Canseco, M. del S.P.; Carrillo, L.T.; Mayoral, L.P.-C.; Treviño, M.V.; Apreza, E.L.; Laguna, R.R. Fiber Optic Sensors: A Review for Glucose Measurement. *Biosens. 2021, Vol. 11, Page 61* **2021**, *11*, 61, doi:10.3390/BIOS11030061.
15. Rivero, D.S.; Urrutia, A.; Ruiz-Zamarreno, C.; Villar, I. Del Advances in Fiber Optic DNA-Based Sensors: A Review. *IEEE Sens. J.* **2021**, *21*, 12679–12691, doi:10.1109/JSEN.2020.3011738.

16. Kumar, S.; Singh, R. Recent optical sensing technologies for the detection of various biomolecules: Review. *Opt. Laser Technol.* **2021**, *134*, 106620, doi:10.1016/J.OPTLASTEC.2020.106620.

17. Massaroni, C.; Zaltieri, M.; Lo Presti, D.; Nicolo, A.; Tosi, D.; Schena, E. Fiber Bragg Grating Sensors for Cardiorespiratory Monitoring: A Review. *IEEE Sens. J.* **2020**, *21*, 14069–14080, doi:10.1109/jsen.2020.2988692.

18. De, M.; Gangopadhyay, T.K.; Singh, V.K. Prospects of photonic crystal fiber for analyte sensing applications: An overview. *Meas. Sci. Technol.* 2020, *31*, 042001.

19. Roriz, P.; Silva, S.; Frazão, O.; Novais, S. Optical Fiber Temperature Sensors and Their Biomedical Applications. *Sensors 2020, Vol. 20, Page 2113* **2020**, *20*, 2113, doi:10.3390/S20072113.

20. Su, H.; Iordachita, I.I.; Tokuda, J.; Hata, N.; Liu, X.; Seifabadi, R.; Xu, S.; Wood, B.; Fischer, G.S. Fiber-Optic Force Sensors for MRI-Guided Interventions and Rehabilitation: A Review. *IEEE Sens. J.* **2017**, *17*, 1952–1963, doi:10.1109/JSEN.2017.2654489.

21. Poeggel, S.; Tosi, D.; Duraibabu, D.; Leen, G.; McGrath, D.; Lewis, E. Optical Fibre Pressure Sensors in Medical Applications. *Sensors 2015, Vol. 15, Pages 17115-17148* **2015**, *15*, 17115–17148, doi:10.3390/S150717115.

22. Dziuda, Ł. Fiber-optic sensors for monitoring patient physiological parameters: a review of applicable technologies and relevance to use during magnetic resonance imaging procedures. *J. Biomed. Opt.* **2015**, *20*, 010901, doi:10.1117/1.JBO.20.1.010901.

23. Falkowski, P.; Lukaszewski, Z.; Gorodkiewicz, E. Potential of surface plasmon resonance biosensors in cancer detection. *J. Pharm. Biomed. Anal.* **2021**, *194*, 113802, doi:10.1016/J.JPBA.2020.113802.

24. Esposito, F.; Srivastava, A.; Sansone, L.; Giordano, M.; Campopiano, S.; Iadicicco, A. Label-Free Biosensors Based on Long Period Fiber Gratings: A Review. *IEEE Sens. J.* **2021**, *21*, 12692–12705, doi:10.1109/JSEN.2020.3025488.

25. Andriukov, B.G.; Lyapun, I.N.; Matosova, E. V.; Somova, L.M. Biosensor technologies in medicine: From detection of biochemical markers to research into molecular targets (review). *Sovrem. Tehnol. v Med.* **2020**, *12*, 70–83, doi:10.17691/stm2020.12.6.09.

26. Presti, D.L.O.; Member, S.; Massaroni, C.; Sofia, C.; Leitão, J.; Domingues, M.D.E.F.; Sypabekova, M.; Barrera, D.; Floris, I.; Massari, L.; et al. Fiber Bragg Gratings for Medical Applications and Future Challenges : A Review. *IEEE Access* **2020**, *8*, 156863–156888.

27. Qu, J.H.; Dillen, A.; Saeys, W.; Lammertyn, J.; Spasic, D. Advancements in SPR biosensing technology: An overview of recent trends in smart layers design, multiplexing concepts, continuous monitoring and in vivo sensing. *Anal. Chim. Acta* 2020, *1104*, 10–27.

28. Zhao, Y.; Tong, R. jie; Xia, F.; Peng, Y. Current status of optical fiber biosensor based on surface plasmon resonance. *Biosens. Bioelectron.* 2019, *142*,

111505.

29. Dinish, U.S.; Beffara, F.; Humbert, G.; Auguste, J.-L.; Olivo, M. Surface-enhanced Raman scattering-active photonic crystal fiber probe: Towards next generation liquid biopsy sensor with ultra high sensitivity. *J. Biophotonics* **2019**, *12*, e201900027, doi:10.1002/JBIO.201900027.

30. Giaquinto, M. (INVITED) Stimuli-responsive materials for smart Lab-on-Fiber optrodes. *Results Opt.* **2021**, *2*, 100051, doi:10.1016/J.RIO.2020.100051.

31. Pisco, M.; Cusano, A. Lab-On-Fiber Technology: A Roadmap toward Multifunctional Plug and Play Platforms. *Sensors 2020, Vol. 20, Page 4705* **2020**, *20*, 4705, doi:10.3390/S20174705.

32. Wang, Q.; Wang, L. Lab-on-fiber: Plasmonic nano-arrays for sensing. *Nanoscale* **2020**, *12*, 7485–7499, doi:10.1039/d0nr00040j.

33. Xiong, Y.; Xu, F. Multifunctional integration on optical fiber tips: challenges and opportunities. *Adv. Photonics* **2020**, *2*, 064001, doi:10.1117/1.AP.2.6.064001.

34. Pissadakis, S. Lab-in-a-fiber sensors: A review. *Microelectron. Eng.* **2019**, *217*, 111105, doi:10.1016/J.MEE.2019.111105.

35. Vaiano, P.; Carotenuto, B.; Pisco, M.; Ricciardi, A.; Quero, G.; Consales, M.; Crescitelli, A.; Esposito, E.; Cusano, A. Lab on Fiber Technology for biological sensing applications. *Laser Photon. Rev.* **2016**, *10*, 922–961, doi:10.1002/LPOR.201600111.

36. Yang, X.; Gong, C.; Liu, Y.; Rao, Y.; Smietana, M.; Gong, Y. Recent Progress in Fiber Optofluidic Lasing and Sensing. *Photonic Sensors* **2021**, *11*, 262–278, doi:10.1007/s13320-021-0622-9.

37. Li, L.; Zhang, Y.; Zhou, Y.; Zheng, W.; Sun, Y.; Ma, G.; Zhao, Y. Optical Fiber Optofluidic Bio-Chemical Sensors: A Review. *Laser Photon. Rev.* **2021**, *15*, 2000526, doi:10.1002/LPOR.202000526.

38. Zhang, L.; Tang, Y.; Tong, L. Micro-/Nanofiber Optics: Merging Photonics and Material Science on Nanoscale for Advanced Sensing Technology. *iScience* **2020**, *23*, 100810, doi:10.1016/J.ISCI.2019.100810.

39. Xiong, C.; Liao, C.; Li, Z.; Yang, K.; Zhu, M.; Zhao, Y.; Wang, Y. Optical Fiber Integrated Functional Micro-/Nanostructure Induced by Two-Photon Polymerization. *Front. Mater.* **2020**, *0*, 369, doi:10.3389/FMATS.2020.586496.

40. Pallarés-Aldeiturriaga, D.; Roldán-Varona, P.; Rodríguez-Cobo, L.; López-Higuera, J.M. Optical fiber sensors by direct laser processing: A review. *Sensors (Switzerland)* **2020**, *20*, 1–37, doi:10.3390/s20236971.

41. Zhao, Y.; Zhao, H.; Lv, R. qing; Zhao, J. Review of optical fiber Mach–Zehnder interferometers with micro-cavity fabricated by femtosecond laser and sensing applications. *Opt. Lasers Eng.* **2019**, *117*, 7–20, doi:10.1016/J.OPTLASENG.2018.12.013.

42. Galeotti, F.; Pisco, M.; Cusano, A. Self-assembly on optical fibers: A powerful nanofabrication tool for next generation "lab-on-fiber" optrodes. *Nanoscale* **2018**, *10*, 22673–22700, doi:10.1039/c8nr06002a.

43. Li, X.; Chen, N.; Zhou, X.; Gong, P.; Wang, S.; Zhang, Y.; Zhao, Y. A review of specialty fiber biosensors based on interferometer configuration. *J. Biophotonics* 2021, *14*, e202100068.

44. Baratto, C.; Faglia, G.; Carletti, L.; De Angelis, C. New Trends in Optical Resonant Bio-Chemical Sensing. *IEEE Sens. J.* **2021**, *21*, 12856–12867, doi:10.1109/JSEN.2021.3055423.

45. Chiavaioli, F.; Janner, D. Fiber Optic Sensing with Lossy Mode Resonances: Applications and Perspectives. *J. Light. Technol.* **2021**, *39*, 3855–3870, doi:10.1109/JLT.2021.3052137.

46. Cennamo, N.; Pesavento, M.; Zeni, L. A review on simple and highly sensitive plastic optical fiber probes for bio-chemical sensing. *Sensors Actuators B Chem.* **2021**, *331*, 129393, doi:10.1016/J.SNB.2020.129393.

47. Li, Y.; Xin, H.; Zhang, Y.; Li, B. Optical Fiber Technologies for Nanomanipulation and Biodetection: A Review. *J. Light. Technol.* **2021**, *39*, 251–262, doi:10.1109/JLT.2020.3023456.

48. Chen, C.; Wang, J. Optical biosensors: An exhaustive and comprehensive review. *Analyst* 2020, *145*, 1605–1628.

49. Zhao, Y.; Zhao, J.; Zhao, Q. Review of no-core optical fiber sensor and applications. *Sensors Actuators, A Phys.* **2020**, *313*, 112160, doi:10.1016/j.sna.2020.112160.

50. Ferreira, M.F.S.; Castro-Camus, E.; Ottaway, D.J.; López-Higuera, J.M.; Feng, X.; Jin, W.; Jeong, Y.; Picqué, N.; Tong, L.; Reinhard, B.M.; et al. Roadmap on optical sensors. *J. Opt. (United Kingdom)* **2017**, *19*, doi:10.1088/2040-8986/aa7419.
